# Two Cdc2 Kinase Genes with Distinct Functions in Vegetative and Infectious Hyphae in *Fusarium graminearum*

**DOI:** 10.1371/journal.ppat.1004913

**Published:** 2015-06-17

**Authors:** Huiquan Liu, Shijie Zhang, Jiwen Ma, Yafeng Dai, Chaohui Li, Xueliang Lyu, Chenfang Wang, Jin-Rong Xu

**Affiliations:** 1 State Key Laboratory of Crop Stress Biology for Arid Areas, College of Plant Protection, Northwest Agriculture and Forestry University, Yangling, Shaanxi, China; 2 Department of Botany and Plant Pathology, Purdue University, West Lafayette, Indiana, United States of America; Wageningen University, NETHERLANDS

## Abstract

Eukaryotic cell cycle involves a number of protein kinases important for the onset and progression through mitosis, most of which are well characterized in the budding and fission yeasts and conserved in other fungi. However, unlike the model yeast and filamentous fungi that have a single Cdc2 essential for cell cycle progression, the wheat scab fungus *Fusarium graminearum* contains two *CDC2* orthologs. The *cdc2A* and *cdc2B* mutants had no obvious defects in growth rate and conidiation but deletion of both of them is lethal, indicating that these two *CDC2* orthologs have redundant functions during vegetative growth and asexual reproduction. However, whereas the *cdc2B* mutant was normal, the *cdc2A* mutant was significantly reduced in virulence and rarely produced ascospores. Although deletion of *CDC2A* had no obvious effect on the formation of penetration branches or hyphopodia, the *cdc2A* mutant was limited in the differentiation and growth of infectious growth in wheat tissues. Therefore, *CDC2A* plays stage-specific roles in cell cycle regulation during infectious growth and sexual reproduction. Both *CDC2A* and *CDC2B* are constitutively expressed but only *CDC2A* was up-regulated during plant infection and ascosporogenesis. Localization of Cdc2A- GFP to the nucleus but not Cdc2B-GFP was observed in vegetative hyphae, ascospores, and infectious hyphae. Complementation assays with chimeric fusion constructs showed that both the N- and C-terminal regions of Cdc2A are important for its functions in pathogenesis and ascosporogenesis but only the N-terminal region is important for its subcellular localization. Among the Sordariomycetes, only three Fusarium species closely related to *F*. *graminearum* have two *CDC2* genes. Furthermore, *F*. *graminearum* uniquely has two Aurora kinase genes and one additional putative cyclin gene, and its orthologs of *CAK1* and other four essential mitotic kinases in the budding yeast are dispensable for viability. Overall, our data indicate that cell cycle regulation is different between vegetative and infectious hyphae in *F*. *graminearum* and Cdc2A, possibly by interacting with a stage-specific cyclin, plays a more important role than Cdc2B during ascosporogenesis and plant infection.

## Author Summary

In the model yeasts and filamentous fungi, *CDC2* is an essential gene that encodes the only CDK essential for mitotic cell cycle progression. However, the wheat scab fungus *F*. *graminearum* contains two *CDC2* orthologs. The *cdc2A* and *cdc2B* deletion mutants had no defects in vegetative growth but deletion of both is lethal. Whereas the *cdc2B* mutant was normal, the *cdc2A* mutant was almost non-pathogenic, indicating that only Cdc2A is essential in infectious hyphae. Cdc2A and Cdc2B differ in subcellular localization and only localization of Cdc2A to the nucleus was increased in cells active in mitosis. Furthermore, *F*. *graminearum* uniquely has two orthologs of Ipl1 Aurora kinase and mutants deleted of orthologs of five essential yeast mitotic kinase genes were viable. However, most of these mutants were significantly reduced in virulence. Overall, our data indicate that *F*. *graminearum* differs from the model fungi in CDK and other key mitotic kinase genes, and cell cycle regulation is different between vegetative and infectious hyphae. This is the first report on two Cdc2 kinases in fungi and they differ in subcellular localization and functions during sexual reproduction and plant infection.

## Introduction

In eukaryotic cells, progression through mitosis and cell cycle is governed by a complex regulatory system. The central components of this system are the cyclin-dependent kinases (CDKs) [1, 2] that are activated by binding to a regulatory cyclin subunit as well as by phosphorylation of a threonine residue adjacent to the kinase active site by the CDK-activating kinase (CAK) [3, 4]. The budding yeast *Saccharomyces cerevisiae* and fission yeast *Schizosaccharomyces pombe* are two model organisms used extensively for cell cycle studies [5]. Both of them have a single CDK gene (*CDC2* or *CDC28*) that is required for all cell cycle transitions [1, 4, 6]. The *cdc2* or *cdc28* null mutant is non-viable [7, 8]. Other members of the CDK family are involved in various cellular processes rather than cell cycle [1]. In addition to CDKs, a number of protein kinases are important for the onset and progression through mitosis, including members of the Aurora, Polo-like, and NimA kinase families as well as kinases involved in checkpoints that regulate entry and exit of mitosis [6, 9–12].

Studies in the budding and fission yeasts have provided insights into the common cell cycle control mechanisms for eukaryotes. Nevertheless, distinctive features and unusual mechanisms of mitosis and cell cycle regulation also are apparent in the two yeast species [4, 5]. For examples, a single cyclin, Cdc13, is sufficient for the mitotic function of Cdc2 in *S*. *pombe*. In *S*. *cerevisiae*, at least four M-cyclins (Clb1-Clb4) are required to orderly activate Cdc28 for progression into mitosis [1]. Furthermore, whereas *S*. *cerevisiae* has a single essential CAK gene *CAK1*, the fission yeast employs two partially redundant CAK systems, the Mcs6-Mcs2 complex and Csk1, to activate Cdc2 [1, 13, 14]. Cak1 is required for the activation of Cdc28 in *S*. *cerevisiae* but Csk1 is not essential for Cdc2 activity in *S*. *pombe*. *KIN28*, the ortholog of *S*. *pombe msc6*, lacks CAK activity in the budding yeast.

Most of the mitotic protein kinase genes identified in *S*. *cerevisiae* or *S*. *pombe* are present in filamentous ascomycetes [15, 16]. Similar to the yeasts, the model filamentous fungi *Aspergillus nidulans* and *Neurospora crassa* have a single *CDC2* ortholog that regulates cell cycle progression and is essential for growth [17, 18]. Interestingly, in a previous study to systematically characterize its kinome [19], we found that *Fusarium graminearum*, a causal agent of wheat and barley head blight disease, contains two putative *CDC2* orthologs that were named *CDC2A* and *CDC2B* here. Interestingly, only the *cdc2A* mutant was defective in sexual reproduction and plant infection [19]. To further characterize the functional divergence of these two *CDC2* orthologs in *F*. *graminearum*, in this study we examined the expression profiles and subcellular localizations of Cdc2A and Cdc2B in different growth and infection stages. Cdc2A and Cdc2B differed in subcellular localization and only Cdc2A is required for cell cycle regulation in infectious hyphae and asci. Functional studies suggested that both the N- and C-terminal regions of Cdc2A are important for its function during ascosporogenesis and pathogenesis but only the N-terminal region is responsible for its subcellular localization. Furthermore, other pivotal mitotic kinases of *F*. *graminearum*, such as CAK and Aurora kinases, also have distinctive features in comparison with those of the budding and fission yeasts. Overall, our data indicate that *F*. *graminearum* differs from the model yeasts and filamentous fungi in CDK and other key mitotic kinase genes, and cell cycle regulation is different between vegetative and infectious hyphae. Cdc2A but not Cdc2B plays a stage-specific role in regulating cell cycle progression during ascospore formation and plant infection.

## Results

### Comparative analysis of two *CDC2* orthologs in *Fusarium* species

**Fig 1:**
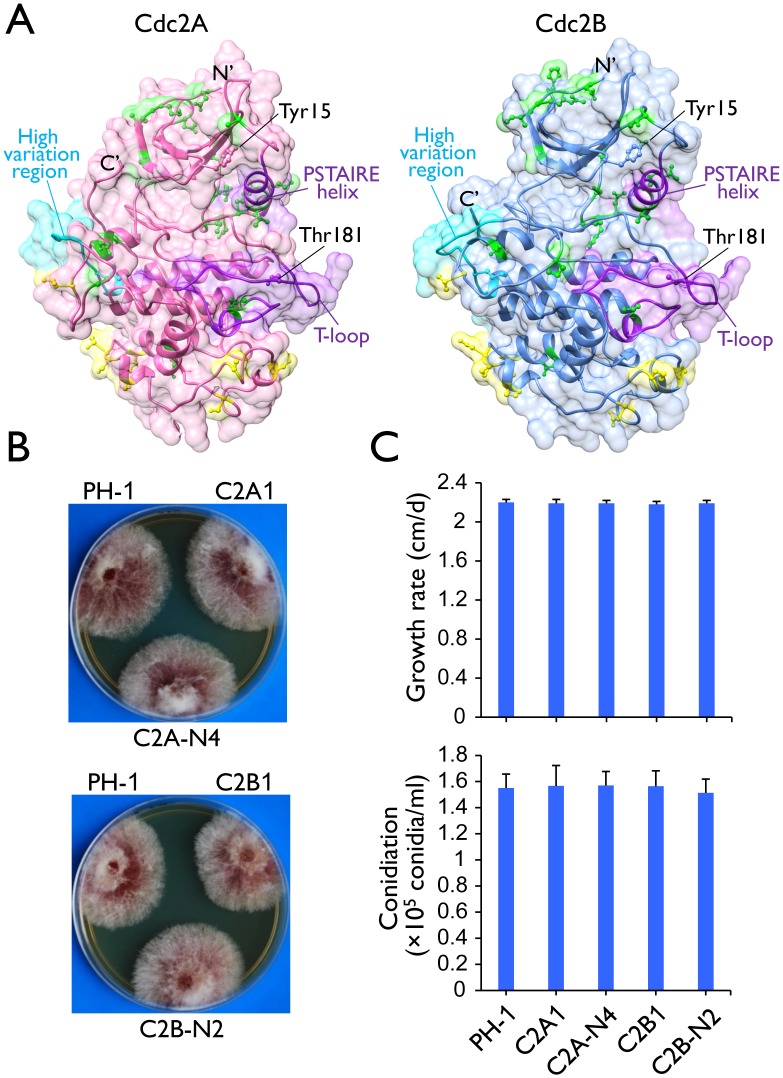
Two Cdc2 co-orthologs in *F*. *graminearum*. (A) Tertiary structures of Cdc2A and Cdc2B predicted by I-TASSER server (http://zhanglab.ccmb.med.umich.edu/I-TASSER/). The N- and C-terminals, the key landmarks including the PSTAIRE helix and the T-loop (activation loop), and the inhibitory (Tyr15) and activating (Thr181) phosphorylation residues [1, 4] are indicated. Yellow and green colors indicate amino acid sites with type I and type II functional divergence between Cdc2A and Cdc2B, respectively. The high sequence variation region between Cdc2A and Cdc2B was also indicated. For detailed residues please see S1 Fig. (B) Colony morphology of the wild type (PH-1), *cdc2A* and *cdc2B* deletion mutants (C2A1 and C2B1), and the complemented transformants (C2A-N4 and C2B-N2). (C) Growth rate and conidiation of PH-1, C2A1, C2B1, C2A-N4, and C2B-N2. Growth rate was measured with race tube cultures (PDA) after incubating at 25°C for 10 days. Conidiation was assayed with 5-day-old CMC cultures. Mean and standard deviation were calculated from data of three independent experiments.

Unlike the model fungi used for cell cycle studies, *F*. *graminearum* has two putative *CDC2* orthologs that were named *CDC2A* (FGSG_08468) and *CDC2B* (FGSG_03132) in this study. The Cdc2A and Cdc2B proteins share 78% identity in amino acid sequences and both have typical CDK structural components, including the PSTAIRE helix, a critical cyclin interface, and the activation T-loop, as well as the inhibitory and activating phosphorylation sites (Fig 1A and S1 Fig). Among all the sequenced Sordariomycetes, only *Fusarium* species, including *F*. *verticillioides*, *F*. *pseudograminearum*, and *F*. *solani*, have two *CDC2* genes. The *F*. *oxysporum* genome contains three *CDC2* orthologs. Phylogenetic analysis revealed that Cdc2A and Cdc2B orthologs from *Fusarium* species clustered together and their phylogeny relationship follows species evolution (S2 Fig). However, the two internal branches leading to the Cdc2B clade were remarkably long (S2 Fig), reflecting a distinctive evolutionary origin of Cdc2B.

Synteny analysis showed that the *CDC2A* locus and its flanking sequences are well conserved in *Fusarium* and other closely related species, including *Trichoderma reesei* (S3 Fig), *Metarhizium acridum*, and *Verticillium dahliae*. In contrast, the flanking sequences of *CDC2B* share only limited homologous regions among *Fusarium* species. There is no detectable similarity between the flanking sequences of *CDC2A* and *CDC2B* in *F*. *graminearum* and other *Fusarium* species. These results together with the Cdc2 phylogeny indicated that *CDC2A* is the original copy in *Fusarium* whereas *CDC2B* is the derived one, possibly by horizontal gene transfer (HGT).

### *CDC2A* and *CDC2B* have redundant functions during vegetative growth

The putative *cdc2A* and *cdc2B* deletion mutants were generated by the split-marker approach in a previous study of the *F*. *graminearum* kinome [19]. For both *CDC2A* and *CDC2B*, three independent deletion mutants (Table 1) were confirmed by Southern blot analysis (S4 Fig) in this study. All three mutants of each gene had the same phenotype although data were presented for only one of them below. The *cdc2A* and *cdc2B* mutants had normal growth rate and colony morphology on PDA (Fig 1B). None of them had obvious defects in conidium morphology and conidiation (Fig 1C). The *cdc2A* and *cdc2B* mutants also were normal in response to oxidative, hyperosmotic, cell wall, and membrane stresses (S5A Fig). Therefore, neither *CDC2A* nor *CDC2B* is essential for conidiation and vegetative growth in *F*. *graminearum*.

**Table 1:** *Fusarium graminearum* strains used in this study.

Strain	Genotype description	Reference
PH-1	The wild-type strain of *F*. *graminearum*	[76]
C2A1	*cdc2A* deletion mutant of PH-1	[19] and this study
C2A2	*cdc2A* deletion mutant of PH-1	[19] and this study
C2A4	*cdc2A* deletion mutant of PH-1	[19] and this study
C2A3	*CDC2A* ectopic integration transformant of PH-1	[19] and this study
C2B1	*cdc2B* deletion mutant of PH-1	[19] and this study
C2B3	*cdc2B* deletion mutant of PH-1	[19] and this study
C2B4	*cdc2B* deletion mutant of PH-1	[19] and this study
C2B2	*CDC2B* ectopic integration transformant of PH-1	[19] and this study
AK2	*Fgcak1* deletion mutant of PH-1	[19] and this study
AK3	*Fgcak1* deletion mutant of PH-1	[19] and this study
AK4	*Fgcak1* deletion mutant of PH-1	[19] and this study
AK1	*FgCAK1* ectopic integration transformant of PH-1	[19] and this study
IP1	*ipl1B* deletion mutant of PH-1	[19]
IP2	*ipl1B* deletion mutant of PH-1	[19]
IP3	*ipl1B* deletion mutant of PH-1	[19]
IP4	*ipl1B* deletion mutant of PH-1	[19]
WE6	*Fgwee1* deletion mutant of PH-1	[19]
RAD1	*Fgrad53* deletion mutant of PH-1	[19]
MEC1	*Fgmec1* deletion mutant of PH-1	[19]
DBF1	*Fgdbf2* deletion mutant of PH-1	[19]
CD15-1	*Fgcdc15* deletion mutant of PH-1	[19]
C2A-N3	*CDC2A*-GFP transformant of C2A1	This study
C2A-N4	*CDC2A*-GFP transformant of C2A1	This study
C2B-N1	*CDC2B*-GFP transformant of C2B1	This study
C2B-N2	*CDC2B*-GFP transformant of C2B1	This study
AK1-2C	*FgCAK1*-GFP transformant of AK2	This study
A-B-1	*CDC2A/2B*-GFP transformant of mutant C2A1	This study
A-B-2	*CDC2A/2B-*GFP transformant of mutant C2A1	This study
A-B-3	*CDC2A/2B*-GFP transformant of mutant C2A1	This study
B-A-1	*CDC2B/2A*-GFP transformant of mutant C2A1	This study
B-A-20	*CDC2B/2A*-GFP transformant of mutant C2A1	This study
A55	*FgCAK1*-3×FLAG & *CDC2A*-GFP transformant of PH-1	This study
A56	*FgCAK1*-3×FLAG & *CDC2A*-GFP transformant of PH-1	This study
D61	*CDC2B*-GFP & *FgCAK1*-3×FLAG transformant of PH-1	This study
D62	*CDC2B*-GFP & *FgCAK1*-3×FLAG transformant of PH-1	This study

Considering the importance of Cdc2/Cdc28 kinase in cell cycle, lack of growth defects in the *cdc2A* and *cdc2B* mutants indicated that these two protein kinase genes have overlapping functions. To test this hypothesis, we transformed the *CDC2A* knockout construct into the *cdc2B* mutant C2B1. After screening over 50 transformants from different transformation experiments, we failed to identify putative double mutants. We also failed to identify *cdc2A cdc2B* double mutants by transforming the *CDC2B* knockout construct into the *cdc2A* mutant C2A1. Although *CDC2A* and *CDC2B* individually are dispensable for growth, deleting both of them appears to be lethal. Therefore, these two Cdc2 orthologs must have redundant functions in cell cycle progression during vegetative growth and asexual reproduction.

### *CDC2A* but not *CDC2B* plays a critical role in ascosporogenesis

**Fig 2:**
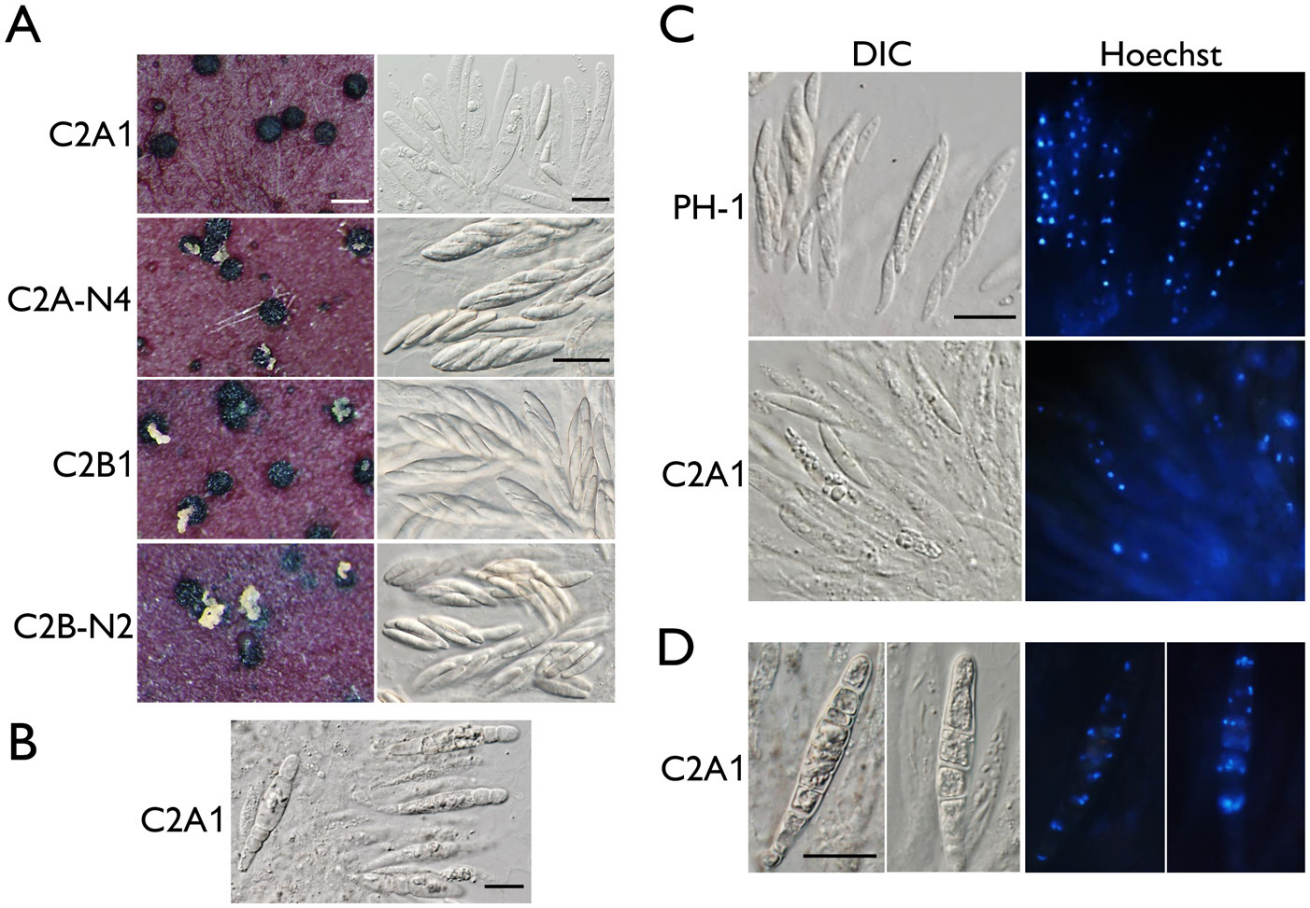
Assays for defects in sexual reproduction in the *cdc2A* and *cdc2B* deletion mutants. (A) Mating cultures of the *cdc2A* and *cdc2B* mutants (C2A1 and C2B1) and corresponding complemented transformants (C2A-N4 and C2B-N2) were examined for cirrhus production on perithecia (left column) and asci or ascospores in cracked perithecia (right column). Whereas the *cdc2B* mutant was normal in sexual reproduction, the *cdc2A* mutant rarely produced mature asci with ascospores. White bar = 1 mm; Black bar = 20 μm. (B) Septated asci formed inside 3-week perithecia of the *cdc2A* mutant (C2A1). Bar = 20 μm. (C) Asci and ascospores released from cracked perithecia of PH-1 and *cdc2A* mutant (C2A1) were stained with Hoechst and examined by differential interference contrast (DIC) or epifluorescence microscopy (Hoechst). Bar = 20 μm. (D) More than one nuclei are observed in each compartment of septated asci produced by the *cdc2A* mutant (C2A1). Bar = 20 μm.

When cultured on carrot agar plates for sexual reproduction, like the wild-type strain, the *cdc2B* mutant produced perithecia with cirrhi of ascospores (Fig 2A) 14 days post-fertilization (dpf). The *cdc2A* mutant was normal in perithecium formation but its perithecia failed to produce ascospore cirrhi 14 dpf or longer (Fig 2A). When perithecia were cracked open, it had fascicles of asci but rarely contained mature ascospores (Fig 2A). Most of the asci formed by the *cdc2A* mutant were aborted in ascosporogenesis and became degenerated. Among the rare *cdc2A* asci with ascospores, none of them had 8 ascospores. In contrast, they tended to contain one or two abnormally large ascospores (Fig 2A). Interestingly, some asci appeared to be septated and contained 4 or more compartments in 3-week or older perithecia produced by the *cdc2A* mutant (Fig 2B). These results suggest that *CDC2A* is important for ascosporogenesis but not the initial ascus development in *F*. *graminearum*. Although they have overlapping functions during vegetative growth and asexual reproduction, the specific requirement for *CDC2A* during ascosporogenesis is not replaceable by *CDC2B*.

When fascicles of asci were stained with Hoechst, the *cdc2A* mutant was significantly reduced in the number of nuclei in comparison with those of PH-1 (Fig 2C). Many asci appeared to be degenerated and lacked nuclei. Nevertheless, four nuclei were observed in some of the rare *cdc2A* ascospores. Therefore, Cdc2A may play a stage-specific role in CDK activities during meiosis and the following mitotic events in developing asci for the production of eight ascospores. In septated asci, each compartment normally contained more than one nuclei (Fig 2D). It is possible that after karyogamy, the diploid nuclei in young asci went into mitosis instead of meiosis, which is similar to the ‘return-to-growth (RTG) phenomenon observed in the *swe1* mutant of *S*. *cerevisiae* [20].

### *CDC2A* is also uniquely required for infectious growth and plant infection

**Fig 3:**
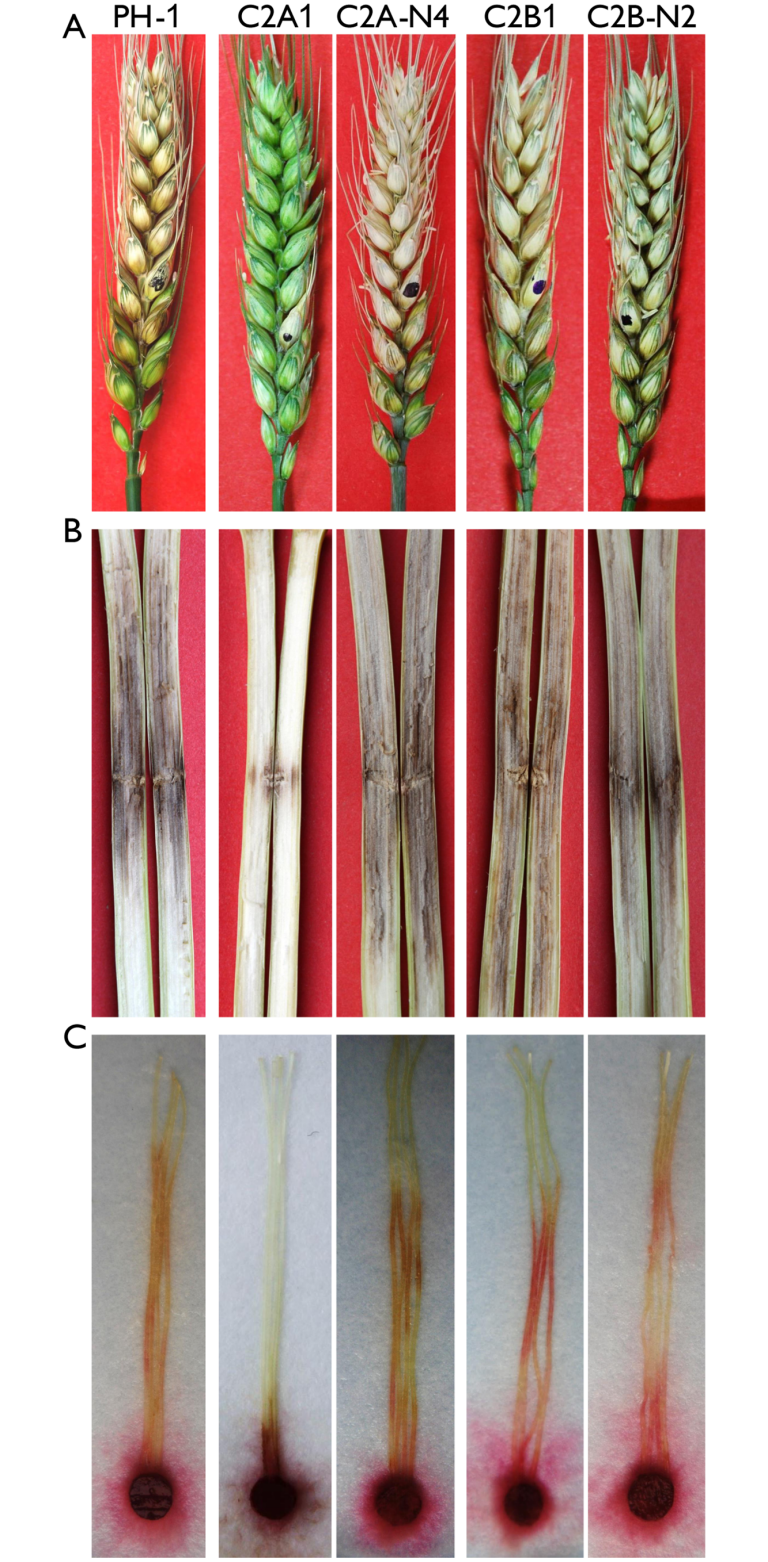
Infection assays with the *cdc2A* and *cdc2B* mutants. (A) Flowering wheat heads were drop-inoculated with conidia of the wild type (PH-1), the *cdc2A* and *cdc2B* mutants (C2A1 and C2B1), and corresponding complemented transformants (C2A-N4 and C2B-N2). Black dots mark the inoculated spikelets. Photographs were taken 14 days post-inoculation (dpi). (B) Eight-week-old corn stalks were punctured with toothpicks dipped in conidia suspensions of the same set of strains. Photographs were taken 14 dpi. (C) Corn silks were inoculated with culture blocks of the same set of strains and examined 6 dpi.

We also assayed the defects of the *cdc2A* and *cdc2B* mutants in plant infection. On flowering wheat heads, the *cdc2B* mutant was as virulent as the wild type. In contrast, the *cdc2A* mutant caused only limited symptoms on the inoculated kernels in infection assays with flowering wheat heads (Fig 3A). On average, the disease index for PH-1 and the *cdc2A* mutant was 13.9 and 1.4, respectively.

To confirm the role of *CDC2A* in virulence, we also conducted infection assays with corn silks and stalks. Whereas the *cdc2B* mutant caused as extensive stalk rot as PH-1 14 days post-inoculation (dpi), the *cdc2A* mutant only caused limited stalk rot at the inoculation sites (Fig 3B). Similar results were obtained in corn silk infection assays. In comparison with PH-1 and the *cdc2B* mutant, the *cdc2A* mutant only caused limited necrosis and discoloration near the inoculation sites (Fig 3C). These results indicate that *CDC2A* but not *CDC2B* plays a critical role in plant infection. Therefore, it is likely that *CDC2A* has a stage-specific role in cell cycle regulation in infectious hyphae.

### The *cdc2A* mutant is defective in infectious growth but not the differentiation of penetration branches

**Fig 4:**
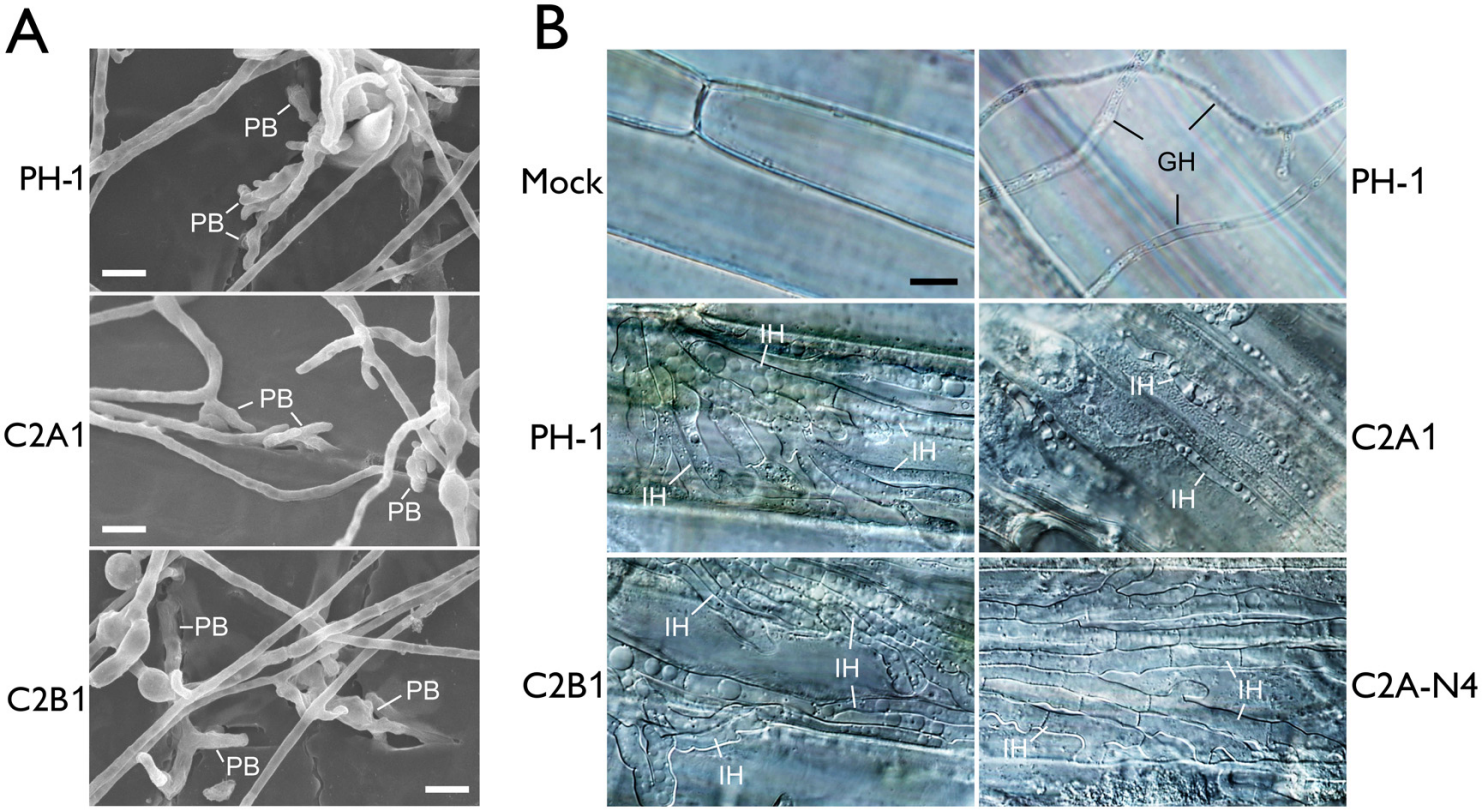
Assays for defects of the *cdc2A* and *cdc2B* mutant in plant infection and colonization. (A) Wheat glumes were inoculated with conidia of the wild type (PH-1) and *cdc2A* and *cdc2B* mutants (C2A1 and C2B1) and examined by SEM 24 h post-inoculation (hpi). PB, penetration branches. Bar = 10 μm. (B) Infectious hyphae (IH) formed by PH-1, C2A1, C2B1, and the complemented *cdc2A*/*CDC2A*-GFP transformant (C2A-N4) inside wheat coleoptile cells at 72 hpi. For comparison, the healthy plant sample inoculated with water (Mock) is shown. The epiphytic Germ tube and Hyphae (GH) is indicated. Bar = 10 μm.

To further characterize the defect of the *cdc2A* mutant in plant infection, we first assayed the formation of penetration branches or infection cushion by scanning electron microscopy (SEM) examination. On wheat lemma surfaces, the *cdc2A* mutant had no obvious defects in the formation of penetration branches (Fig 4A). Therefore, *CDC2A* and *CDC2B* likely have overlapping functions in the differentiation of initial penetration structures on plant surfaces.

Because DON is an important virulence factor in *F*. *graminearum* [21], we then assayed DON production in both rice grain cultures [22] and liquid trichothecene biosynthesis induction (TBI) medium [23]. The *cdc2A* mutant produced the same level of DON compared to the wild type and *cdc2B* mutant (S1 Table). It also formed the thick, bulbous hyphal compartments as efficiently as the wild type and *cdc2B* mutant in the liquid TBI culture (S5B Fig). Therefore, the defect of *cdc2A* mutant in plant infection is not related to DON production.

*F*. *graminearum* is known to form infectious hyphae that have distinct hyphal morphology from vegetative hyphae inside wheat coleoptiles [24]. We then examined the differentiation and growth of infectious hyphae in the *cdc2A* and *cdc2B* mutants. Similar to the wild type, the *cdc2B* mutant developed extensive infectious hyphae inside wheat coleoptile cells 72 hpi (Fig 4B). Under the same conditions, it is difficult to observe infectious hyphae in most of the samples inoculated with the *cdc2A* mutant. Rare infectious hyphae formed by the *cdc2A* mutant in wheat coleoptiles had restricted growth and limited branches (Fig 4B). These results indicate that *CDC2A* but no *CDC2B* is important for infectious growth in plant tissues. Therefore, *CDC2A* must play a more critical role than *CDC2B* in regulating cell cycle progression in infectious hyphae although they have overlapping functions in vegetative hyphae.

### *CDC2A* is up-regulated during plant infection and sexual reproduction

**Fig 5:**
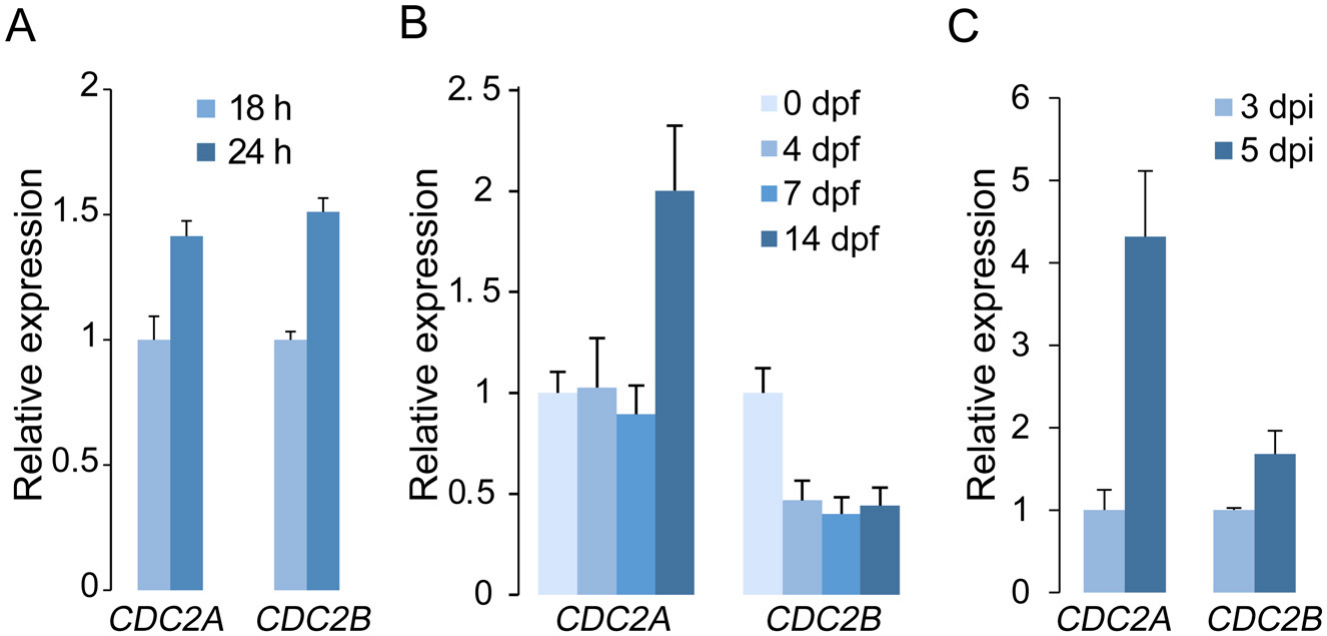
Expression levels of *CDC2A* and *CDC2B* assayed by qRT-PCR. (A) Relative expression levels of *CDC2A* and *CDC2B* in germlings and vegetative hyphae. The expression level of each gene in 18 h germlings was arbitrarily set to 1. (B) Relative expression levels of *CDC2A* and *CDC2B* in mating cultures at different day-post-fertilization (dpf). Their expression level at 0 dpf was arbitrarily set to 1. (C) Relative expression levels of *CDC2A* and *CDC2B* at 3 and 5 days post-inoculation (dpi). Their expression level at 3 dpi was arbitrarily set to 1. Error bar indicates standard deviation (SD) calculated from data of three replicates.

When assayed by qRT-PCR, transcripts of *CDC2A* and *CDC2B* were detectable in germ tubes, vegetative hyphae, mating cultures, and infected wheat heads (Fig 5). Therefore, the lack of detectable phenotypes in the *cdc2B* mutant is not related to its stage-specific expression. Interestingly, *CDC2B* expression was reduced after carrot agar cultures were fertilized. The expression of *CDC2A* was not significantly affected in early stages of sexual reproduction but up-regulated at 14 dpf (Fig 5B). During plant infection, both *CDC2A* and *CDC2B* were up-regulated at 5 dpi in comparison with their expression at 3 dpi. However, the expression of *CDC2A* increased much more significantly than that of *CDC2B* (Fig 5C). These expression data are consistent with the importance of Cdc2A during ascosporogenesis and plant infection.

### Distinct localization of Cdc2A and Cdc2B

To determine their subcellular localization, the *CDC2A-* and *CDC2B*-GFP fusion constructs generated by gap repair were transformed into the *cdc2A* and *cdc2B* mutants, respectively. For each gene, at least two GFP fusion transformants (Table 1) were obtained. The *cdc2B*/*CDC2B*-GFP transformant, similar to the *cdc2B* mutant, had the wild-type phenotype. The two *cdc2A*/*CDC2A*-GFP transformants, C2A-N3 and C2A-N4 (Table 1), were normal in growth, sexual reproduction (Fig 2), and plant infection (Fig 3), indicating that the *CDC2A*-GFP fusion fully complemented the *cdc2A* mutant. Therefore, deletion of *CDC2A* is directly responsible for the defects observed in the *cdc2A* mutant.

**Fig 6:**
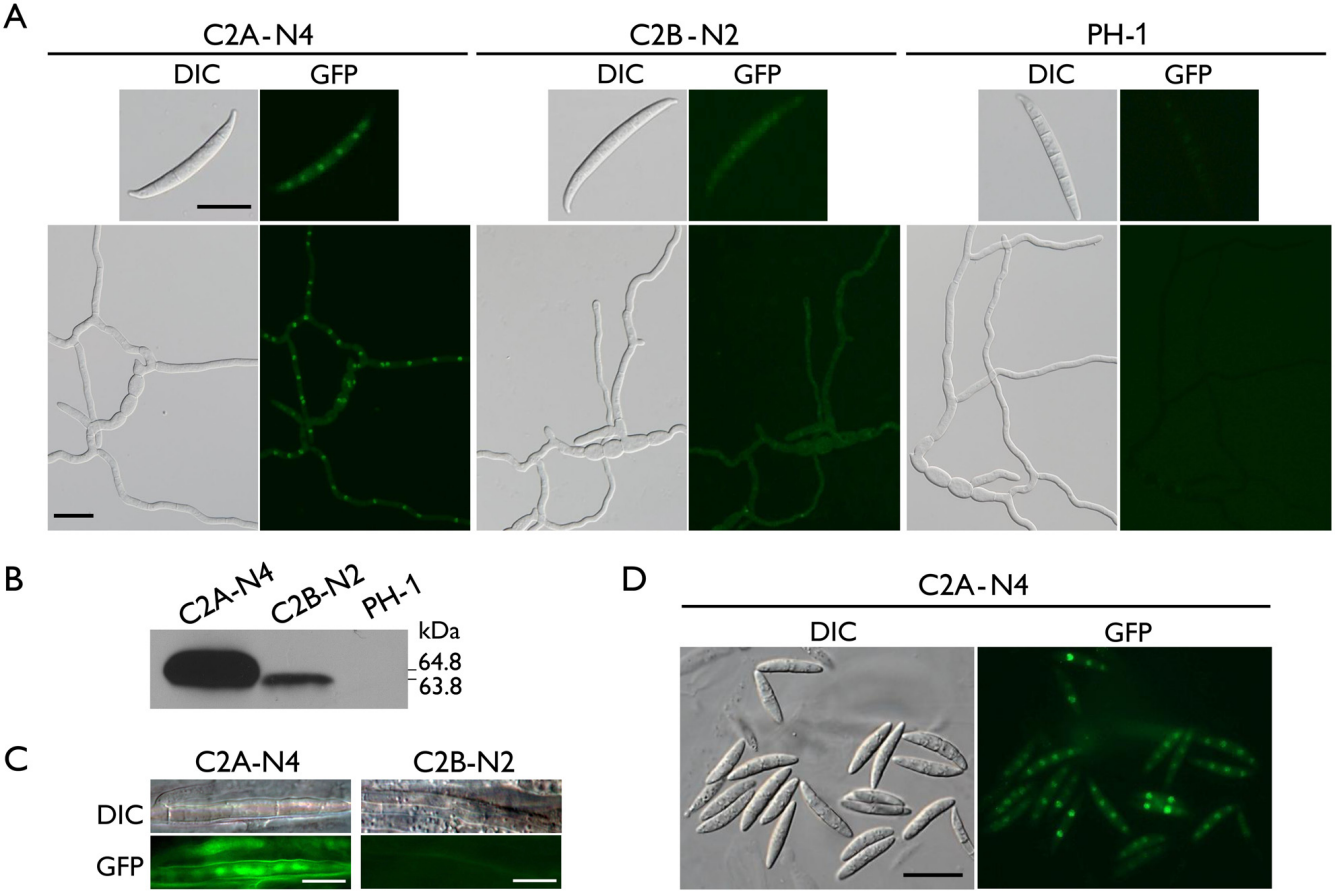
Expression and subcellular localization of Cdc2A-GFP and Cdc2B-GFP fusion proteins. (A) Conidia and germlings (12 h) of the *CDC2A*-GFP (C2A-N4) and *CDC2B*-GFP (C2B-N2) transformants were examined by differential interference contrast (DIC) and epifluorescence (GFP) microscopy. The wild type PH-1 is shown as a negative control. Bar = 20 μm. (B) Western blots of Cdc2A-GFP (64.8 kDa) and Cdc2B-GFP (63.8 kDa) expression in C2A-N4 and C2B-N2 transformants, respectively, detected with the anti-GFP antibody. PH-1 is shown as a negative control. (C) Infectious hyphae formed by the *CDC2A*-GFP and *CDC2B*-GFP transformants inside wheat coleoptiles 48 hpi. GFP signals were observed in the nucleus in the *CDC2A-*GFP but not *CDC2B-*GFP transformant. Bar = 10 μm. (D) Ascospores of the *CDC2A*-GFP transformant. The young ascospore contains two nuclei had stronger GFP signals in the nucleus than the other mature ascospores with four nuclei. Bar = 20 μm.

In both *CDC2A-* and *CDC2B*-GFP transformants, GFP signals were observed in conidia and vegetative hyphae, which is consistent with the fact that these two genes are constitutively expressed. In general, GFP signals appeared to be stronger in the *CDC2A*-GFP transformant than in the *CDC2B-*GFP transformant (Fig 6A). Western blot analysis confirmed the lower expression level of Cdc2B-GFP fusion compared to Cdc2A-GFP (Fig 6B). The Cdc2A-GFP fusion proteins were distributed throughout the cell but significantly enriched in the nucleus (Fig 6A and S6 Fig). In comparison with un-germinated conidia, young germ tubes had stronger GFP signals in the nucleus (Fig 6A), indicating that localization of Cdc2A to the nucleus was increased when nuclear division becomes active. In contrast, no significant changes in the subcellular localization of GFP signals were observed in the *CDC2B-GFP* transformant in conidia and germ tubes (Fig 6A and S6 Fig). GFP signals in the nucleus were faint in the *cdc2B*/*CDC2B*-GFP transformant.

### Localization of Cdc2A to the nucleus in infectious hyphae

Because the *cdc2A* mutant was defective in infectious hyphae growth, we also examined the localization of Cdc2A-GFP during colonization of wheat coleoptiles. In wheat samples inoculated with the *CDC2A-*GFP transformant, GFP signals were observed in the nucleus of infectious hyphae developed inside coleoptile cells (Fig 6C). In contrast, we failed to observe GFP signals above the background in infectious hyphae formed by the *CDC2B-*GFP transformant in wheat coleoptiles (Fig 6C). It is possible that both up-regulation of *CDC2A* and its localization to the nucleus in infectious hyphae are related to the importance of *CDC2A* during invasive growth.

### Enhanced localization of Cdc2A to the nucleus in developing ascospores

Because one of the major defects of the *cdc2A* mutant was in ascosporogenesis, we also examined the localization of Cdc2A-GFP during sexual reproduction. In the *CDC2A-GFP* transformant, GFP signals were observed in both the cytoplasm and nucleus in mature, four-celled ascospores, with stronger signals in the nucleus (Fig 6D). However, in comparison with mature ascospores, GFP signals in the nucleus appeared to be stronger in two or three-celled developing ascospores that need one more round of mitosis to become four-celled (Fig 6C). This observation was confirmed by examined over one hundred of microscopic fields in two independent experiments. These results indicate that localization of Cdc2A to the nucleus may be increased in young ascospores undergoing mitosis, which is consistent with the observation of stronger GFP signals in germ tubes than in conidia (Fig 6A).

### Both the N- and C-terminal regions are important for the function of Cdc2A in pathogenesis

**Fig 7:**
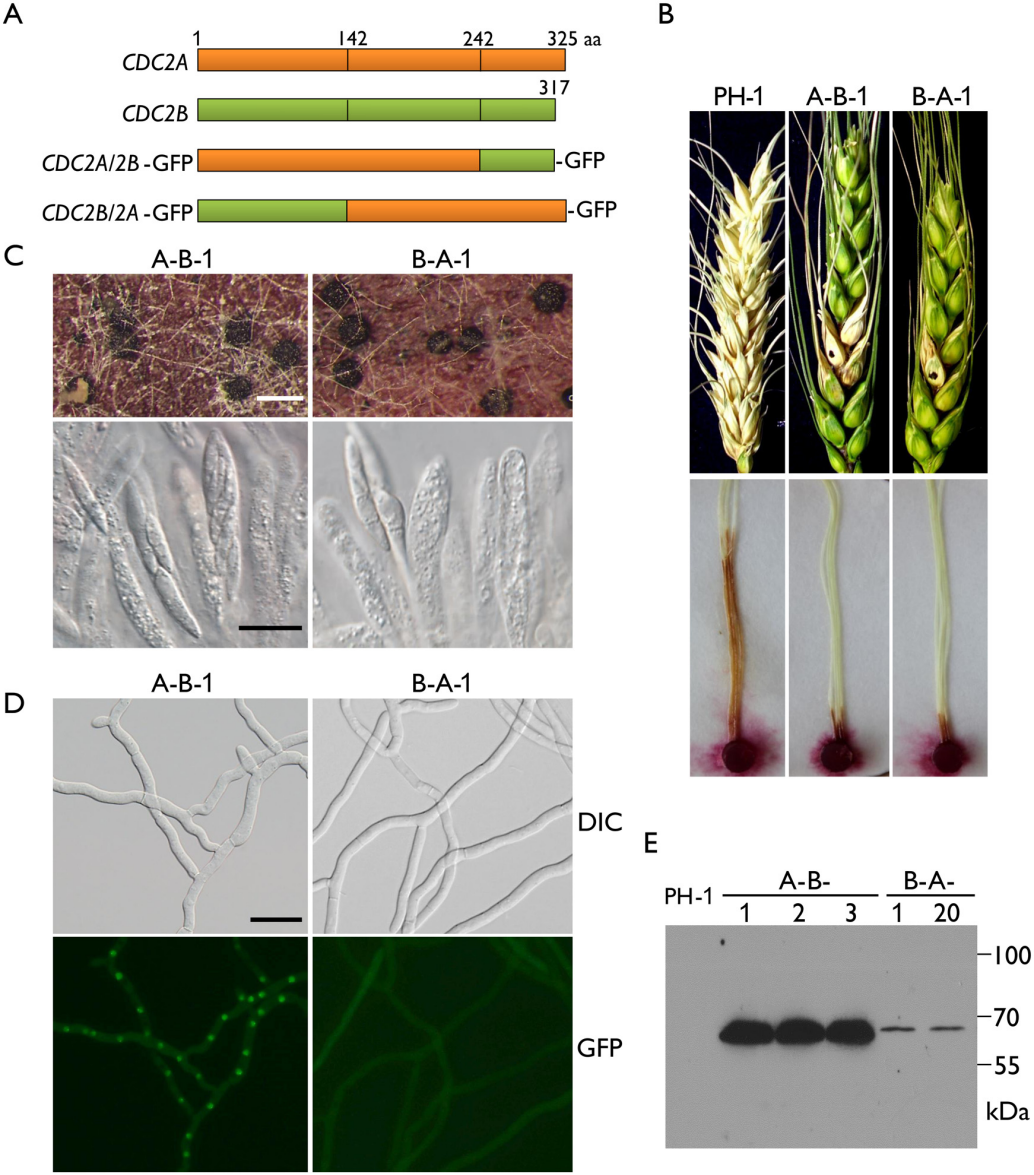
Defects in plant infection and sexual reproduction and subcellular localization of *CDC2A/2B-* and *CDC2B/2A-*GFP chimeric transformants. (A) Schematic diagrams of the *CDC2A* and *CDC2B* genes and the *CDC2A/2B-* and *CDC2B/2A-*GFP fusion constructs. Orange and green boxes indicate the coding regions of *CDC2A* and *CDC2B*, respectively. Black vertical lines in boxes mark out the N- and C-terminal regions of Cdc2A and Cdc2B. For detailed residues please see S1 Fig. (B) Flowering wheat heads were drop-inoculated with conidia of the wild type (PH-1) and the *CDC2A/2B-*GFP and *CDC2B/2A-*GFP transformants (A-B-1 and B-A-1). Black dots mark the inoculated spikelets. Photographs were taken 14 dpi. Corn silks were inoculated with culture blocks of the same set of strains and examined 6 dpi. (C) Perithecia formed by transformants A-B-1 and B-A-1 were examined for ascospore cirrhi (upper row) and asci or ascospores in cracked perithecia (lower row). White bar = 1 mm; Black bar = 20 μm. (D) Subcellular localization of the Cdc2A/2B-GFP and Cdc2B/2A-GFP proteins. Germlings (12 h) of transformants A-B-1 and B-A-1 were examined by differential interference contrast (DIC) and epifluorescence (GFP) microscopy. Bar = 20 μm. (E) Western blot analysis of GFP fusion expression in three *CDC2A/2B-*GFP (A-B-1, A-B-2, and A-B-3) and two *CDC2B/2A-*GFP (B-A-1 and B-A-12) transformants detected with the anti-GFP antibody.

Although they are highly similar in sequences and structural components, Cdc2A and Cdc2B have a number of significant type I and type II functional divergence sites [25, 26] between them (Fig 1A and S1 Fig). Most of these sites distributed in the N-terminal region adjacent to the cyclin binding [1] or inhibitory phosphorylation sites and the C-terminal region (Fig 1A and S1 Fig). To identify the Cdc2A region responsible its functional specificity, we generated the *CDC2A* ^1-242^ *-CDC2B* ^243-317^-GFP (*CDC2A/2B*-GFP) and *CDC2B* ^1-142^ *-CDC2A* ^143-325^-GFP (*CDC2B/2A*-GFP) chimeric constructs by replacement of the N- or C-terminal regions of *CDC2A* with those of *CDC2B* (Fig 7A), and transformed them into the *cdc2A* mutant. For each construct, at least two transformants with same phenotypes were obtained (Table 1). In infection assays, similar to the original *cdc2A* mutant, the *CDC2A/2B-*GFP and the *CDC2B/2A-*GFP transformants caused only limited symptoms on flowering wheat heads and corn silks (Fig 7B). These results indicate that both *CDC2A/2B-*GFP and *CDC2B/2A-*GFP fusion constructs failed to complement the defects of the *cdc2A* mutant in pathogenesis. Therefore, it is likely that both N- and C-terminal regions of *CDC2A* are important for its stage-specific functions in cell cycle regulation during plant infection.

When assayed for sexual reproduction, the *CDC2B/2A* transformant was similar to the original *cdc2A* deletion mutants in ascosporogenesis defects (Fig 7C). Interestingly, the *CDC2A/2B* transformant was partially recovered in ascospore formation. Although it still produced much fewer ascospores than the wild type, asci with 8-ascospores were produced by the *CDC2A/2B* transformant (Fig 7C). These data indicate that the N-terminal region of *CDC2A* plays a more critical role in ascosporogenesis than its C-terminal region.

### The N-terminal region of Cdc2A is responsible for its localization to the nucleus

Because Cdc2A but not Cdc2B had distinct localization to the nucleus, we examined the subcellular localization of *CDC2A/2B*-GFP and *CDC2B/2A*-GFP fusion constructs. Similar to the *CDC2A-*GFP transformant, the *CDC2A/2B*-GFP transformant had stronger GFP signals in the nucleus (Fig 7D). However, localization of GFP fusion proteins to the nucleus was not observed in the *CDC2B/2A*-GFP transformant, which is similar to the *CDC2B*-GFP transformant (Fig 7D). In addition, we noticed that GFP signals were stronger in the *CDC2A/2B*-GFP transformant than in the *CDC2B/2A*-GFP transformant (Fig 7D), which was confirmed by western blot analysis (Fig 7E). These results indicate that the N-terminal region of *CDC2A* was responsible for its subcellular localization.

### The inhibitory phosphorylation level was higher for Cdc2A than for Cdc2B

**Fig 8:**
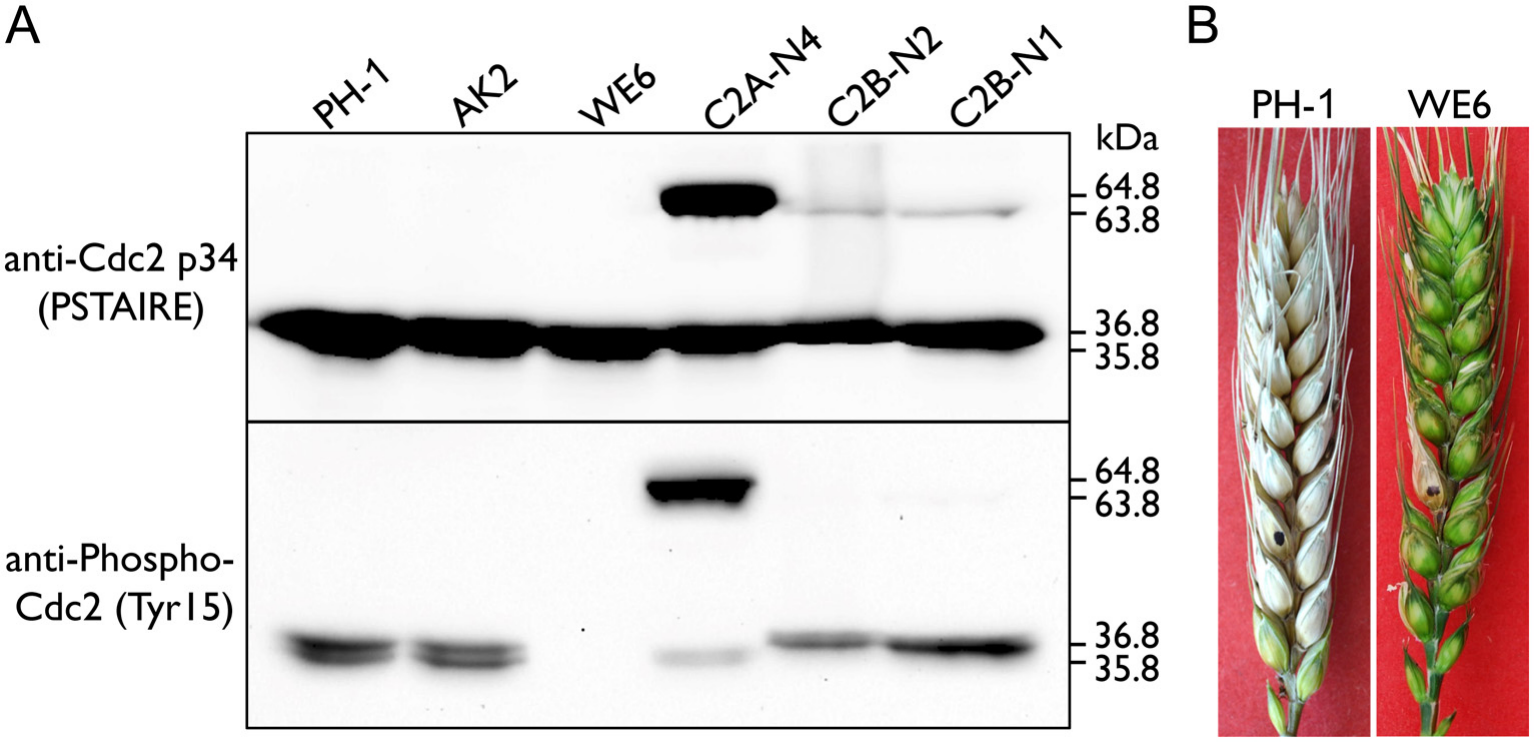
Assays for inhibitory phosphorylation of Cdc2A and Cdc2B and defects of the *Fgwee1* deletion mutants in plant infection. (A) Total proteins were isolated from the wild-type (PH-1), *CDC2A*-GFP (C2A-N4) and *CDC2B*-GFP (C2B-N1 and C2B-N2) transformants, and the *Fgcak1* (AK2) and *Fgwee1* (WE6) deletion mutants. The Cdc2A, Cdc2B, Cdc2A-GFP and Cdc2B-GFP proteins were detected with the Cdc2 p34 (PSTAIRE) antibody. The phosphorylation of them at tyrosine 15 was detected with the Phospho-Cdc2 (Tyr15) antibody. The band of Cdc2A (36.8 kDa), Cdc2B (35.8 kDa), Cdc2A-GFP (64.8 kDa) and Cdc2B-GFP (63.8 kDa) is indicated. (B) Flowering wheat heads inoculated with the wild type (PH-1) and *Fgwee1* mutant (WE6). Disease symptoms were examined 14 dpi.

Inhibitory phosphorylation at the well-conserved Tyr15 is known to affect CDK activation and nuclear localization [27]. In *Ustilago maydis* inhibitory phosphorylation of Cdk1 is required for conjugation tube formation and plant penetration [28]. Therefore, we used the anti-Tyr15 antibody [29, 30] to assay inhibitory phosphorylation levels of Cdc2A and Cdc2B in *F*. *graminearum*. The expression level of Cdc2A in the two *cdc2B*/*CDC2B*-GFP transformants (C2B-N1 and C2B-N2) was comparable to that of Cdc2B in the *cdc2A*/*CDC2A*-GFP transformant (C2A-N4). However, the inhibitory phosphorylation level of Cdc2A was obviously higher than that of Cdc2B (Fig 8A). This result was confirmed by at least three independent phosphorylation assays.

Inhibitory phosphorylation of CDKs is mediated by protein kinases orthologous to *S*. *pombe* Wee1 and *S*. *cerevisiae* Swe1 [1]. In the mutant deleted of *FgWEE1* (FGSG_10228), the inhibitory phosphorylation of Cdc2A and Cdc2B was completely blocked (Fig 8A). The *Fgwee1* deletion mutant was aborted in ascus or ascospore development [19]. Like the *cdc2A* mutant, it also was defective in plant infection (Fig 8B). Therefore, lack of inhibitory phosphorylation by FgWee1 likely resulted in improper regulation of Cdc2A and Cdc2B activities and defects in cell cycle and cytokinesis regulation in *F*. *graminearum*.

### FgCak1 interacts with both Cdc2A and Cdc2B

**Fig 9:**
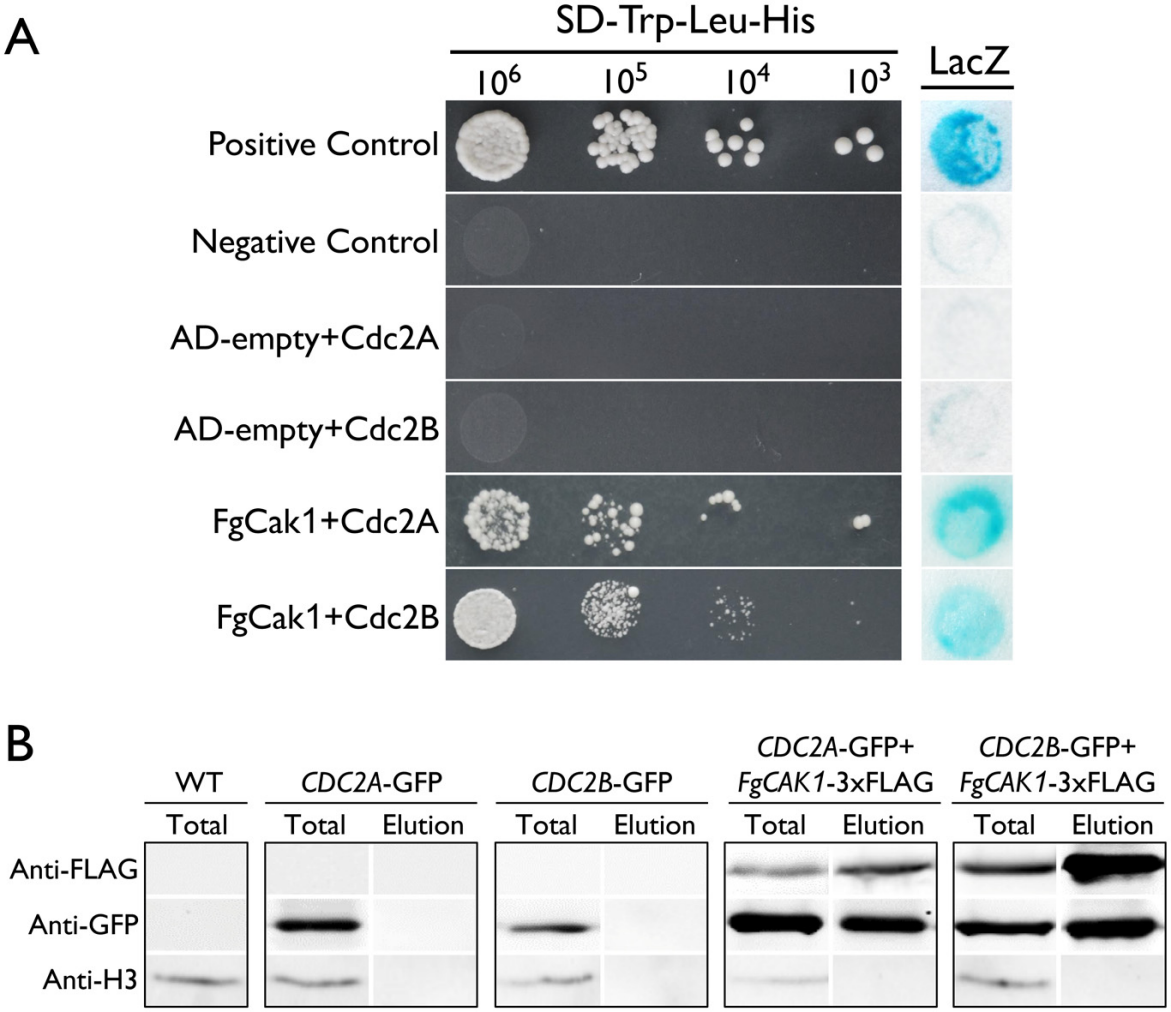
Yeast two-hybrid and co-immunoprecipitation (co-IP) assays for the interaction of FgCak1 with Cdc2A and Cdc2B. (A) Different concentrations of yeast cells (cells/ml) of the transformants expressing the marked bait and prey constructs (labelled on the left) were assayed for growth on SD-His plates. The positive and negative controls are shown. The auto-activation controls AD-empty+Cdc2A and AD-empty+Cdc2B are also shown. The same set of yeast transformants was assayed for β-galactosidase (LacZ) activities. (B) Co-IP assays. Immunoblots of total proteins (Total) and proteins eluted from the anti-FLAG M2 beads (Elution) from *F*. *graminearum* transformants expressing the GFP fusion construct alone or co-expressing GFP and 3xFLAG fusion constructs as labeled on the top. Western blots were detected with anti-FLAG, anti-GFP or anti-Histone H3 antibody. Total proteins isolated from the wild-type (WT) PH-1 were included as the control.

In addition to binding with cyclins, the complete activation of Cdc28 requires phosphorylation by the Cak1 kinase at the conserved T residue in the T-loop in *S*. *cerevisiae* [3, 4]. A single *CAK1* ortholog (FGSG_04947) was identified in *F*. *graminearum*. In yeast two-hybrid assays, FgCak1 interacted with both Cdc2A and Cdc2B (Fig 9A). To confirm their interactions by co-IP assays, the *FgCAK1*-3xFLAG construct was co-transformed with the *CDC2A*- or *CDC2B-GFP* fusion construct into PH-1. In the resulting transformants (Table 1), the Cdc2A-GFP or Cdc2B-GFP band could be detected with an anti-GFP antibody in total proteins and proteins eluted from anti-FLAG beads (Fig 9B). These results indicate that both Cdc2A and Cdc2B interacted with FgCak1.

### The *Fgcak1* deletion mutant is defective in ascosporogenesis and pathogenesis

**Fig 10:**
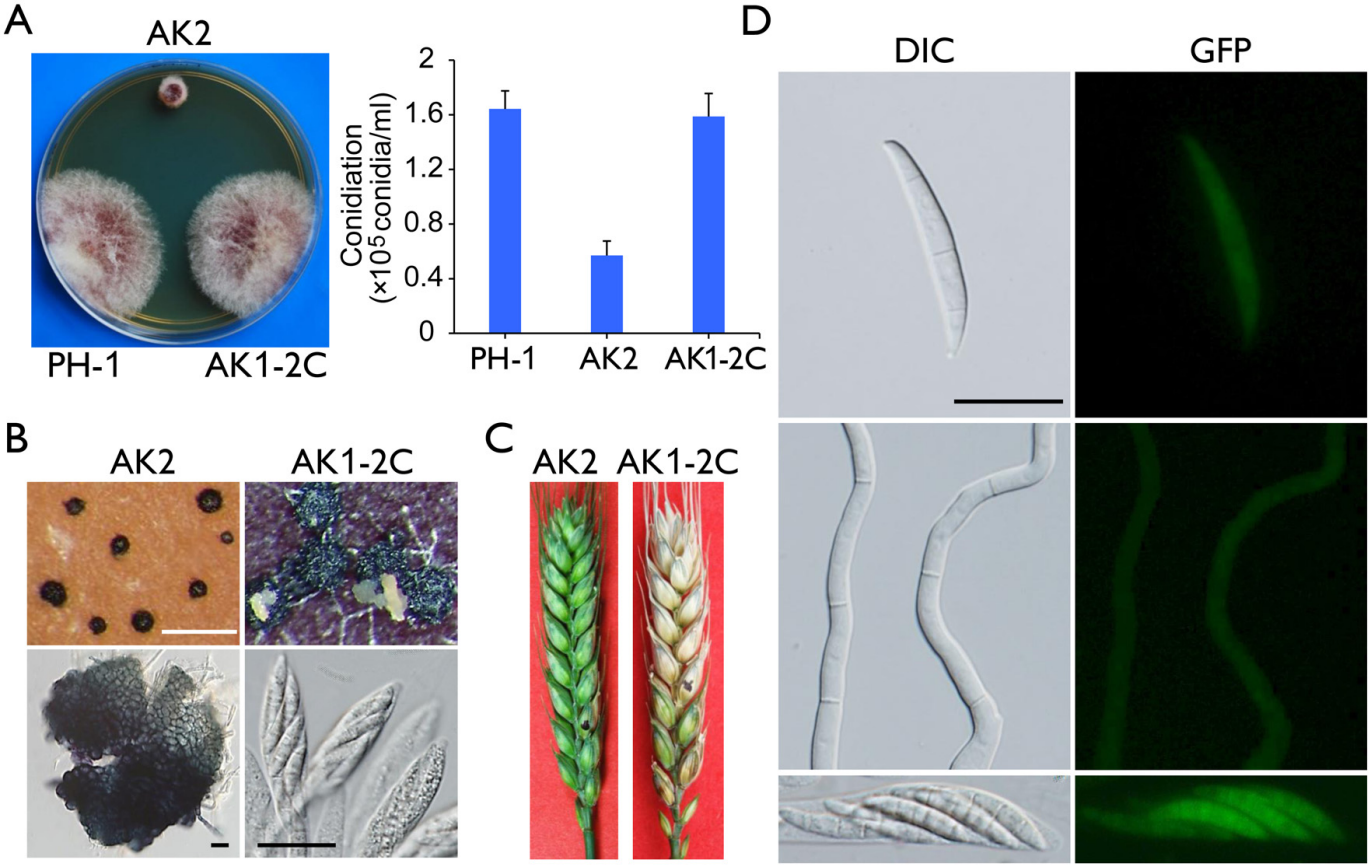
Defects of the *Fgcak1* mutant in vegetative growth, sexual reproduction, and plant infection and subcellular localization of FgCak1-GFP fusion protein. (A) Colony morphology and conidiation of the wild type (PH-1), *Fgcak1* mutant (AK2), and complemented transformant (AK1-2C). Mean and standard deviation were calculated from data of three independent experiments. (B) Mating culture of the *Fgcak1* mutant and corresponding complemented transformant were examined for perithecium formation and cirrhus production (upper row) and asci or ascospores (lower row). White bar = 1 mm; Black bar = 20 μm. (C) Infection assays with flowering wheat heads. Disease symptoms were examined 14 dpi. (D) Subcellular localization of the FgCak1-GFP protein. Conidia, germlings (12 h), and ascospores in asci of the *FgCAK1*-GFP transformant (AK1-2C) were examined by differential interference contrast (DIC) and epifluorescence (GFP) microscopy. Bar = 20 μm.

The *Fgcak1* deletion mutants generated in a previous study [19] were confirmed by Southern blot analysis (S4 Fig) in this study. Unlike the *cdc2A* and *cdc2B* mutants, the *Fgcak1* mutant was significantly reduced in growth rate and conidiation (Fig 10A). On carrot agar plates, it produced perithecia that were smaller than those of PH-1 and the *cdc2A* and *cdc2B* mutants and lacked asci or ascospores (Fig 10B). In addition, The *Fgcak1* mutant failed to cause disease symptoms in infection assays with flowering wheat heads (Fig 10C), suggesting that, unlike *CDC2A*, *FgCAK1* is essential for plant infection and sexual reproduction. It is possible that FgCak1 is involved in the activation of both Cdc2A and Cdc2B during ascosporogenesis and pathogenesis in *F*. *graminearum*.

### Localization of FgCak1 to the cytoplasm

Because FgCak1 interacts with both Cdc2A and Cdc2B, we also examined its subcellular localization. The *FgCAK1*-GFP fusion construct under the control of its native promoter was transformed into the *Fgcak1* mutant AK2. The resulting *Fgcak1*/*FgCAK1*-GFP transformant AK1-2C was normal in growth and conidiation (Fig 10A), sexual reproduction (Fig 10B), and plant infection (Fig 10C), suggesting that fusion with GFP had no adverse effects on FgCak1 function. In transformant AK1-2C, weak GFP signals were observed in the cytoplasm in conidia, germ tubes, and ascospores (Fig 10D), which is consistent with the localization of Cak1 in the budding yeast [31, 32].

### One putative Cdc2-cyclin is up-regulated during plant infection

Based on the phenotypes of the *cdc2A* and *cdc2B* mutants, it is likely that *F*. *graminearum* has one or more Cdc2A-specific cyclin during plant infection and sexual reproduction. The *F*. *graminearum* genome has 17 putative cyclin genes. Based on their orthologs in the budding or fission yeast, three of them, FGSG_00941, FGSG_01291, and FGSG_07132, were predicted to be Cdc2-cyclins (S7A Fig). In comparison with other filamentous fungi, *F*. *graminearum* has one additional *PCL8/PCL10*-like cyclin gene FGSG_07737 (S7A Fig) that lacks a distinct ortholog in the closely related *Fusarium* species. Based on microarray data available at the PLEXdb database (www.plexdb.org), the expression of all three Cdc2-cyclin genes was down-regulated by 3 days-post-fertilization (dpf) but became up-regulated after 4 dpf (S7B Fig). The expression level of FGSG_07132 was higher at 7 dpf than at 1 dpf, suggesting that it may be important for ascosporogenesis. During barley infection, the expression of FGSG_01291 was up-regulated after 24 hpi, which is the time of infection cushion formation and differentiation of infectious hyphae. Its expression continued to increase until 7 dpi, suggesting that FGSG_01291 plays a more critical role than other Cdc2-cyclins during infectious growth. The expression of the extra cyclin in *F*. *graminearum*, FGSG_07737, was relative low during sexual reproduction and decreased during barley infection (S7B Fig). These data indicated that FGSG_07132 and FGSG_01291 may function as *CDC2A*-specific cyclins during sexual reproduction and plant infection, respectively.

## Discussion

CDKs are the central regulators of eukaryotic cell cycle. Although the model yeasts and filamentous fungi used for cell cycle studies have only one mitotic CDK responsible for regulating cell cycle transitions, *F*. *graminearum* and other *Fusarium* species have two *CDC2* co-orthologs. The *CDC2B* sequences from *Fusarium* species form a monophyletic clade and their relationship is consistent with the species phylogeny [33]. However, the *CDC2B* clade clusters specifically with *F*. *solani CDC2A* rather than forms a sister group to the entire *CDC2A* clade (S2 Fig), indicating that *CDC2B* was generated after the divergence of *F*. *solani* from the last common ancestor of the other three species. The phylogeny of *F*. *solani CDC2B* with that of the other three *Fusarium* species suggests that the last common ancestor of *F*. *graminearum*, *F*. *oxysporum*, and *F*. *verticillioides* obtained *CDC2B* from *F*. *solani* or its closely related species, likely by horizontal gene transfer (HGT). Recently, it has been shown that horizontal transfer of genes is prevalent in *Fusarium* species [33, 34].

Cdc2A and Cdc2B share 78% identity in amino acid sequences and likely have similar functions in cell cycle regulation. However, significant type I and type II functional divergence [25, 26] between Cdc2A and Cdc2B was identified (S1 Fig). In addition, the type I functional divergence between Cdc2 proteins of non-*Fusarium* species (S2 Fig) and Cdc2B, but not Cdc2A, was significant (p = 0.001). Therefore, *CDC2A* may maintain the primary function of ancestral *CDC2* copy whereas *CDC2B* may have gained novel or modified functions [35]. This inference is consistent with the fact that accelerated sequence evolution occurred in the two ancestral branches leading to *CDC2B* clade, possibly due to the relaxed purified selection and/or positive selection after HGT.

The *CDC2* ortholog in the model yeasts and filamentous fungi is an essential gene [1, 4, 6, 17, 18], reflecting its key regulatory role in cell cycle. However, deletion of either *CDC2A* or *CDC2B* had no obvious defects in vegetative growth and conidiation in *F*. *graminearum*. The *cdc2A* and *cdc2B* deletion mutants also had no detectable difference with the wild type in response to different environmental stresses. Nevertheless, deletion of both of them appears to be lethal. These results indicate that *CDC2A* and *CDC2B* must have redundant functions in mitotic cell cycle progression during vegetative growth. The absence of Cdc2A can be fully rescued by Cdc2B and *vice versa*, which is consistent with their highly conserved amino acid sequences. Cdc2 is known to form heterodimers with cyclins but not with other CDKs in the model yeast and filamentous fungi, which have a single *CDC2* gene. In the budding yeast, paralogs often interact with each other [36], which may facilitate the evolution of new functions. It is possible that *CDC2B* has evolved to control mitotic cell cycle under specific environmental or physical conditions. Unfortunately, the *cdc2B* deletion mutant had no detectable phenotype.

Unlike their overlapping functions in vegetative growth and asexual reproduction, *CDC2A* and *CDC2B* play different roles in sexual reproduction. Whereas the *cdc2B* mutant was normal, the *cdc2A* mutant was defective in ascosporogenesis. Most of the asci formed by the *cdc2A* mutant were aborted in ascospore development. We failed to observe 8-spored asci and rarely observed four-celled ascospores (Fig 2). In *F*. *graminearum*, a number of mutants are known to be defective in sexual reproduction [19, 37–41]. For example, mutants blocked in any one of the MAPK pathways are female sterile and defective in perithecium formation [19, 39]. The *zif1* and *mat1-1-2* mutants produced smaller perithecia that are blocked in ascus development [40, 41]. Among all the protein kinase genes characterized in *F*. *graminearum*, the *Fgwee1* mutant had similar defects with the *cdc2A* mutant in ascosporogenesis but it produced smaller perithecia [19]. In *S*. *cerevisiae*, Swe1 phosphorylates and inhibits Cdc28 although it is phosphorylated and activated by Cdc28 [42]. They form a stable Swe1-Cdc28 complex to maintain Cdc28 in the inhibited state and play a critical role in controlling the transition into mitosis. In *F*. *graminearum*, Cdc2A and its downstream targets during sexual reproduction may be specifically required for ascospore formation after meiosis but not functionally related to the development of ascogenous hyphae and perithecia.

*CDC2A* also differs from *CDC2B* in their functions during plant infection. Whereas the *cdc2B* mutant was as virulent as the wild type, the *cdc2A* mutant caused only limited necrosis in some of the inoculated kernels. Likely the *cdc2B* mutant, the *cdc2A* mutant was normal in stress responses, DON production, and the development of penetration branches. However, the *cdc2A* but not *cdc2B* was defective in invasive growth. Infection hyphae formed by the *cdc2A* mutant had only limited growth and rarely branched. Therefore, Cdc2A may play an essential in regulating cell cycle progression in infectious hyphae and this function of Cdc2A is not replaceable by Cdc2B. Because *CDC2B* is constitutively expressed, including in infected plant tissues, Cdc2B may have diverged from *CDC2A* and lost or reduced significantly in its mitotic kinase activity in infectious hyphae after HGT.

Proper regulation of cell cycle and cytokinesis has been shown to be important for plant infection in *M*. *oryzae* and *U*. *maydis* [43–45]. In *M*. *oryzae*, infectious and vegetative hyphae differ significantly in morphology. The bulbous infectious hyphae are somewhat similar to pseudohyphae formed by yeast species [46]. In *F*. *graminearum*, infectious hyphae with distinct morphology also are formed inside plant cells [24, 47]. Therefore, the regulation of cell cycle progression may be different between infectious hyphae and vegetative hyphae grown in cultures. Our data supported this hypothesis because *CDC2A* and *CDC2B* have overlapping functions in cell cycle during vegetative growth but only *CDC2A* is required for infectious growth. Consistent with this observation, the expression level of *CDC2A* but not that of *CDC2B* was increased during plant infection. The expression level of *CDC2A* was also increased during sexual reproduction. In nature, sexual reproduction is closely related to plant infection in *F*. *graminearum* because perithecia are formed by hyphae grown in plant debris and ascospores released from perithecia formed on plant debris serve as the primary inoculum.

The subcellular localization of Cdc2/Cdc28 is dynamic and depends on its interacting partners. In the fission yeast, Cdc2 specifically localizes to the nucleus [48]. In the budding yeast, Cdc28 localizes primarily in the cytoplasm but is accumulated in the nucleus in late G1 cells [27, 49]. In *A*. *nidulans*, NimX localizes predominantly to the nucleus [50] In *F*. *graminearum*, Cdc2A mainly localized to the nucleus and its nuclear localization was enhanced in cells undergoing mitosis. In contrast, Cdc2B is present throughout the cell in conidia, germ tubes, and ascospores. The difference in the localization of Cdc2A and Cdc2B may be related to the difference in their interacting proteins and functions, illustrating the functional diversification of these two CDKs. Nevertheless, when the C-terminal region of *CDC2A* was replaced with that of *CDC2B*, the resulting *CDC2A/2B*-GFP transformant has stronger GFP signals in the nucleus. But it is still defect in plant infection and only partially recovered in ascospore formation. These results suggest that nuclear localization of Cdc2A is not critical for infection and sexual development.

Most of the type I and type II functional divergence sites are located in the N- and C-terminal regions of Cdc2A and Cdc2B (S1 Fig). Consistent with this observation, both the N- and C-terminal regions of Cdc2A are important for its specific role in pathogenesis and ascosporogenesis. The N-terminal region contains the cyclin-binding domain and inhibitory phosphorylation sites [1]. In the budding yeast, binding to cyclins and phosphorylation are known to affect the subcellular localization of Cdc28 [27]. In this study, we found that the N-terminal region of Cdc2A is responsible for its localization to the nucleus (Fig 7). Our results also showed that the inhibitory phosphorylation level of Cdc2A was slightly different from Cdc2B (Fig 8). In *U*. *maydis*, inhibitory phosphorylation of Cdk1 is important for plant infection [28]. In *F*. *graminearum*, the *Fgwee1* mutant also was defective in plant infection. Therefore, it is likely that nuclear localization and functional specificity of Cdc2A is related to its activation by inhibitory phosphorylation or cyclin-binding.

Consistent with the weaker GFP signals, the expression levels of *CDC2B*-GFP fusion proteins was significantly lower than that of *CDC2A*-GFP under the control of native promoter (Figs 6B and 8A). While under the control of the same *CDC2A* promoter, the expression levels of *CDC2B/2A*-GFP was also significantly lower than that of *CDC2A/2B*-GFP (Fig 7E). These results suggest that the N-terminal region of *CDC2B* determined its protein level. However, no significant difference was found between the levels of Cdc2B and that of either *CDC2A* or *CDC2A*-GFP (Fig 8A). It is likely that fused with the GFP protein significantly increased the degradation rate of Cdc2B but not Cdc2A.

Both Cdc2A and Cdc2B interact with Cak1, which is encoded by the only CDK kinase gene in *F*. *graminearum*. In *S*. *cerevisiae*, *CAK1* is an essential gene for cell division [51]. In *F*. *graminearum*, deletion of *FgCAK1* is not lethal. The *cak1* mutant shared similar defects with the *cdc2A* mutant in plant infection, indicating that Cak1 may be responsible for the activation of Cdc2A in infectious hyphae. However, deletion of *CAK1* resulted in a significant reduction in growth rate and conidiation. Most likely, Cak1 is involved in the activation of both Cdc2A and Cdc2B for hyphal growth *in vitro* and asexual reproduction. Nevertheless, unlike the *cdc2A cdc2B* double mutant, the *cak1* mutant is viable. In *F*. *graminearum*, Cdc2A and/or Cdc2B may have sufficient residual CDK activities after binding with CDK-cyclins in the absence of FgCak1.

The *F*. *graminearum* genome has 17 putative cyclin genes that are conserved in other Sordariomycetes. Although it contains two Cdc2 co-orthologs, *F*. *graminearum* has no additional Cdc2-cyclins in comparison with *N*. *crassa*, *A*. *nidulans*, and yeasts. One of three putative Cdc2-cyclin genes FGSG_01291 is distinctly up-regulated during plant infection. FGSG_01291 is an ortholog of the *nimE* in *A*. *nidulans* [52]. Increased copy number of nimE can lead to increased accumulation of inhibitory tyrosine phosphorylated Cdc2 [52]. Considering the higher level of inhibitory phosphorylation for Cdc2A, it is likely that FGSG_01291 may uniquely or preferentially interact with Cdc2A. Interestingly, *F*. *graminearum* has one additional *PCL8/PCL10*-like gene, FGSG_07737 that lacks distinct orthologs in other filamentous fungi. It will be interesting to determine whether the putative cyclin gene has novel functions in mitotic cell cycle in *F*. *graminearum*.

Because it differs from the model yeasts in Cdc2 and Cak1 functions, we also analyzed the evolutionary conservation and divergence of other mitotic kinases in *F*. *graminearum*. In comparison with *S*. *cerevisiae* and *S*. *pombe*, *F*. *graminearum* has at least one ortholog of each mitosis related protein kinase genes (S2 Table). One unique feature of *F*. *graminearum* is that it has two Aurora kinase genes, FGSG_06959 (named *IPL1A*) and FGSG_02399 (named *IPL1B*) (S8A Fig). In mammalian cells, Aurora kinases function as mitotic regulators and play a crucial role in cellular division by controlling chromatid segregation [53]. The budding and fission yeasts have a single Aurora kinase gene that regulates the completion of mitotic events and is essential for viability [54–56]. All the sequenced Sordariomycetes, including *F*. *verticillioides*, *F*. *oxysporum*, and *F*. *solani*, have a single Aurora-like gene (S8A Fig). In mammalian cells, Aurora-A and Aurora-B have distinct subcellular localization and function [57–60]. However, when the G198 residue of Aurora-A was replaced with the equivalent N142 residue of Aurora-B, Aurora-A^G198N^ had similar subcellular localization and functions with Aurora-B [61]. In *F*. *graminearum*, at the position equivalent to G198 of Aurora-A, Ipl1A has a G residue but Ipl1B has an S residue (S8B Fig). It is possible that Ipl1A and Ipl1B function similarly to Aurora-A and Aurora-B.

Overall, our studies with *CDC2A* and *CDC2B* indicate that *F*. *graminearum* may differ from *S*. *cerevisiae* and other model fungi in the regulatory mechanism of mitotic cell cycle. Consistent with this observation, *F*. *graminearum* has two putative Aurora kinase genes that regulate chromatid segregation. Furthermore, we found that a number of putative mitotic kinase genes also may differ in functions between *F*. *graminearum* and *S*. *cerevisiae*. Similar to *CDC2* and *CAK1*, the *DBF2/DBF20*, *CDC15*, *MEC1*, and *RAD53* genes are essential in *S*. *cerevisiae*. In *F*. *graminearum*, the *Fgdbf2*, *Fgcdc15* and *Fgmec1* mutants were viable but significantly reduced in virulence [19]. The *KIN3* ortholog was essential in *F*. *graminearum* but the yeast *kin3* deletion mutant is viable [62]. These observations further indicate that *F*. *graminearum* differs from the model yeasts in the function of several mitotic kinase genes. Like in other plant pathogens [43–45], cell cycle progression may be regulated differently in vegetative and infectious hyphae of *F*. *graminearum*.

## Materials and Methods

### Strains, culture conditions, and phenotype assays

The wild-type strain PH-1 of *F*. *graminearum* and its derived transformants used in this study are listed in Table 1. All strains were routinely cultured on PDA plates at 25°C for growth and in carboxymethyl cellulose (CMC) medium for conidiation as described [63]. Growth rate was measured with race tube cultures (PDA) after incubating at 25°C for 10 days. Conidiation was assayed with 5-day-old CMC cultures. The experiments for growth rate and conidiation were totally repeated for 3 times with 3 repetitions for each time. For DNA extraction, vegetative hyphae were harvested from 2-day-old YEPD (1% yeast extract, 2% peptone, 2% glucose) cultures. For sexual reproduction, aerial hyphae of 7-day-old carrot agar cultures were pressed down with 300 μl of 0.1% Tween 20 and incubated under black light [64]. Perithecium formation and cirrhi production were assayed after incubation at 25°C for 2 weeks. For plant infection assays, freshly harvested conidia were re-suspended to 10^5^ spores/ml and used to inoculate flowering wheat heads of cultivar Xiaoyan22 or Norm and stalks of 8-week-old corn plants of cv. Pioneer 2375 as described [41]. Wheat spikelets with typical symptoms were examined 14 days post-inoculation (dpi) and stalk rot symptoms were examined after splitting corn stalks longitudinally along the inoculation site 14 dpi. DON production was assayed with rice grain cultures [22] and liquid trichothecene biosynthesis induction (TBI) media containing 5 mM arginine as previously described [23, 65] with minor modification.

### Targeted deletion *CDC2A* in the *cdc2B* mutant and *vice versa*

The *CDC2A* and *CDC2B* gene replacement constructs were generated with the split-marker approach using the neomycin resistance gene (*NEO* ^*R*^) as the selectable marker. Protoplasts of the *cdc2A* and *cdc2B* mutants were prepared and transformed with the *CDC2B* and *CDC2A* gene replacement constructs as described previously [21, 39]. For transformant selection, hygromycin B (Calbiochem, La Jolla, CA) and geneticin (Sigma-Aldrich, St. Louis, MO) were added to the final concentration of 250 and 150 mg/ml, respectively [66].

### Generation of *CDC2A*-, *CDC2B*-, and *FgCAK1*-GFP fusion constructs and transformants

To generate the GFP fusion constructs, genomic fragments containing the promoter and coding region of the target genes were amplified and cloned into pFL2 vector by the yeast gap repair approach [67]. The resulting in-frame GFP fusion constructs of *CDC2A*, *CDC2B*, and *FgCAK1* were confirmed by DNA sequencing and transformed into protoplasts of the corresponding mutants. The resulting transformants harboring the fusion construct were identified by PCR and further confirmed by the presence of GFP signals. GFP signals in conidia, germ tubes, ascospores and infectious hyphae were observed with a BX51 Olympus epifluorescence microscope or an Olympus FV1000 confocal microscope. Nuclei were stained with Hoechst 33258 (Beyotime, China).

### Generation of *CDC2A*-*CDC2B* chimeric constructs and transformants

Based on the sequence alignment of Cdc2 orthologs (S1 Fig), we divided Cdc2A and Cdc2B into the N- and C-terminal regions as depicted in Fig 7A and S1 Fig. We generated *CDC2A/B* and *CDC2B/A* chimeric alleles under the control of the *CDC2A* promoter by replacement of the N- or C-terminal regions of *CDC2A* with those of *CDC2B* by the yeast GAP repair approach [67, 68], respectively. The resulting chimeric *CDC2A/B*-GFP and *CDC2B/A*-GFP constructs were verified by sequencing analysis and transformed into the *cdc2A* mutant. The resulting *cdc2A CDC2A/2B* or *cdc2A CDC2B/2A* transformants were confirmed by PCR and observation of GFP signals.

### Assays for penetration branches and infectious hyphae

For penetration branches observation, glumes and lemmas were collected from inoculated wheat spikelets 24 h post-inoculation (hpi), and fixed with 4% glutaraldehyde in 0.1 M phosphate buffer (pH 6.8) overnight at 4°C [69]. After dehydration in a series of 30, 50, 70, 80, 90, and 100% acetone, the samples were coated with gold-palladium and examined with a JEOL 6360 scanning electron microscope (SEM) as described [69]. For assaying infectious hyphae, three-day-old seedlings of wheat cultivar Norm were inoculated as described [56]. In brief, the top 2 to 3 mm of the coleoptiles were removed with a razor blade and the wounded seedlings were wrapped in 4 mm X 1 cm cotton strips soaked with *F*. *graminearum* conidia (10^5^ spores/ml). After inoculation, the seedlings were grown at 25°C in a growth chamber with 95% humidity and examined at 48 or 72 hpi with a BX51 Olympus epifluorescence microscope. The experiments were repeated at least 3 times. In each experiment, we examined at least ten samples for each strain.

### Assays for the expression levels of *CDC2A* and *CDC2B*

RNA samples were isolated from germlings, vegetative hyphae, mating cultures, and infected wheat heads with the TRIzol reagent (Invitrogen, Carlsbad, CA) as described [41]. First-strand cDNA was synthesized with the Fermentas 1st cDNA synthesis kit (Hanover, MD) following the instructions provided by the manufacturer. Relative expression levels of *CDC2A* and *CDC2B* were assayed by qRT-PCR with the *FgTUB2* beta-tubulin gene of *F*. *graminearum* [65] as the internal control. For each sample, at least three independent biological replicates were analyzed to calculate mean and standard deviation.

### Yeast two-hybrid assays

The interactions of FgCak1 with Cdc2A and Cdc2B were assayed with the Matchmaker yeast two-hybrid system (Clontech, Mountain View, CA). The ORFs of *CDC2A* and *CDC2B* were amplified from first-strand cDNA of PH-1 synthesized as described [70] and cloned into pGBKT7 (Clontech) as the bait constructs. The ORF of *FgCAK1* was amplified and cloned into pGADT7 as the prey construct. The resulting bait and prey vectors were co-transformed in pairs into yeast strain AH109 (Clontech). To check for auto-activation, the bait construct of *CDC2A* and *CDC2B* were co-transformed with empty pGADT7 vector. The resulting transformants were then assayed for growth on synthetic dropout (SD) medium lacking tryptophan, leucine, and histidine (SD-Trp-Leu-His) and β-galactosidase activities as described [71].

### Co-IP assays

The *CDC2A-* and *CDC2B-*GFP fusion constructs were generated by cloning genomic fragments containing the open reading frame (ORF) of these two genes into pFL2 by the yeast gap repair approach [67, 68]. Similar approaches were used to generate the 3xFLAG fusion constructs for the *FgCAK1* and *CDC2B* genes using the PFL7 vector. The resulting fusion constructs were confirmed by sequencing analysis and transformed in pairs into PH-1. The expression of both transforming vectors was analyzed by PCR and western blot analysis. Total proteins were isolated from strains expressing both transforming constructs and incubated with anti-Flag M2 beads (Sigma-Aldrich, St. Louis, MO) as described [19]. Western blots of total proteins and proteins eluted from anti-Flag M2 beads were detected with anti-GFP (Roche, Indianapolis, IN), anti-FLAG (Sigma-Aldrich), and anti-Histone H3 (Sigma-Aldrich) antibodies as described [72].

### Phosphorylation assays with Cdc2A and Cdc2B

Total proteins were isolated in the presence of phosphatase inhibitor cocktail (Sigma-Aldrich) from 18 h germlings grown in liquid complete medium (CM) cultures, separated on a 12.5% SDS-PAGE, and transferred to nitrocellulose membranes as described [67]. For western blot analysis, the expression of Cdc2A and Cdc2B was detected with the Cdc2 p34 (PSTAIRE) antibody (Santa Cruz Biotechnology, Inc.). The inhibitory phosphorylation level of Cdc2 proteins was detected with the Phospho-Cdc2 (Tyr15) antibody (Cell Signaling Technology, Danvers, MA).

### Sequence comparison and phylogenetic analysis

Multiple alignments of protein sequences were constructed with COBALT (www.ncbi.nlm.nih.gov/tools/cobalt) and manually modified. The analysis of type I and type II functional divergence was performed with the Diverge 3.0 software [73]. Maximum likelihood (ML) phylogenies were estimated with PhyML3.0 [74] assuming 8 categories of γ-distributed substitution rate and SPRs algorithms. For phylogeny of protein sequences, the best-fit model for each datasets selected by ProtTest2.4 [75] was used. The reliability of internal branches was evaluated based on SH-aLRT supports.

### Accession numbers

Cyclin-dependent protein kinase Cdc2A (Gene ID: FGSG_08468, GenBank accession no.: XP_011320297.1), Cyclin-dependent protein kinase Cdc2B (FGSG_03132, XP_011322636.1), Cyclin-dependent kinase-activating kinase FgCak1 (FGSG_04947, XP_011323418.1), Aurora kinase Ipl1A (FGSG_06959, XP_011326636.1), Aurora kinase Ipl1B (FGSG_02399, XP_011318314.1), DNA damage response protein kinase FgRad53 (FGSG_00433, XP_011316100.1), Mitosis entry checkpoint protein kinase FgMec1 (FGSG_13318, XP_011320055.1), Transcription and stress response protein kinase FgDbf2 (FGSG_08635, XP_011320108.1), Mitotic exit network protein kinase FgCdc15 (FGSG_10381, XP_011319350.1), M phase inhibitor protein kinase FgWee1 (FGSG_10228, XP_011319176.1), three putative Cdc2-cyclins (FGSG_00941, XP_011316682.1; FGSG_07132, XP_011326840.1; FGSG_01291, XP_011317073.1), *PCL8/PCL10*-like cyclin (FGSG_07737, XP_011327546.1).

## Supporting Information

S1 Fig Multiple sequence alignment of Cdc2A and Cdc2B. The ancestral sequence reconstructed by the FASTML Server (http://fastml.tau.ac.il/) was shown above as the reference. Residues different from the reference are highlighted in color. The PSTAIRE helix and the T-loop are key landmark regions of Cdc2. ‘#’ and ‘*’ symbols mark the inhibitory (Y15) and activating (T181) phosphorylation sites, respectively. ‘$’ and ‘%’ symbols indicate amino acid sites with type I (highly conserved in one co-ortholog but variable in the other) and type II (highly conserved within both co-orthologs but diverged between them) functional divergence [73] at p > 0.6. Red vertical lines mark the N- and C-terminal regions of Cdc2A and Cdc2B as depicted in Fig 7A.
(PDF)

S2 Fig The maximum likelihood tree of Cdc2 orthologs. The phylogenetic tree was constructed with the protein sequences of catalytic domains. Each branch was marked with the p-values of approximate likelihood ratios (SH-aLRT). Branches with p-values less than 0.5 have been collapsed. Scale bar corresponds to 0.1 amino acid substitutions per site. *F*. *oxysporum* has many lineage-specific genomic regions [33] and is the only fungus with three Cdc2 orthologs.
(PDF)

S3 Fig Gene structure and synteny of *CDC2A* and *CDC2B*. (A) Synteny of the *CDC2A* and *CDC2B* loci and their 40-kb flanking sequences. Collinear orthologous relationships are represented by gray bands. *Fusarium oxysporum* shares a high sequence identity with *F*. *verticillioides* in this region. (B) Intron positions in the *CDC2A* and *CDC2B* genes. Dashed lines mark the homologous region. Fg, *Fusarium graminearum*; Fo, *F*. *oxysporum*; Fv, *F*. *verticillioides*; Fs, *F*. *solani*; Tr, *Trichoderma reesei*.
(PDF)

S4 Fig Southern blot analysis with the *cdc2A*, *cdc2B*, *Fgcak1*, and *ipl1B* deletion mutants. (A) The split-marker approach used to replace the target gene with the hygromycin phosphotransferase (*hph*) cassette. Probe A and probe B are PCR products amplified with primers 5F/6R and H852/H850, respectively. (B) Southern blots of restriction enzyme-digested genomic DNA of PH-1 (WT) and mutants hybridized with probe A (left) and probe B (right). The restriction enzyme used was marked.
(PDF)

S5 Fig Colony and cell morphology of the *cdc2A* and *cdc2B* mutants. (A) Responses of the *cdc2A* and *cdc2B* mutants to oxidative, hyperosmotic, membrane, and cell wall stresses. Four-day old colonies formed by the wild type (PH-1) and *cdc2A* (C2A1) or *cdc2B* (C2B1) deletion mutants on PDA with 0.05% H_2_O_2_, 0.7 M NaCl, 0.01% SDS, or 200 mg/ml Congo Red. (B) Hyphal morphology of PH-1 and the *cdc2A* and *cdc2B* mutants in trichothecene biosynthesis induction (TBI) liquid culture after 72 h of incubation at 25°C.
(PDF)

S6 Fig Subcellular localization of Cdc2A-GFP and Cdc2B-GFP fusion proteins. Germlings (8 h) of the *CDC2A*-GFP (C2A-N4) and *CDC2B*-GFP (C2B-N2) transformants were examined by differential interference contrast (DIC) and confocal microscopy after staining with Hoechst. Bar = 20 μm.
(PDF)

S7 Fig Phylogeny and expression profile of three putative Cdc2-cyclins and one extra *PCL8/PCL10*-like cyclin during plant infection and sexual reproduction. (A) The maximum likelihood tree of orthologs of three putative Cdc2-cyclins FGSG_01291, FGSG_07132, and FGSG_00941 and one extra *PCL8/PCL10*-like cyclin FGSG_07737. The phylogenetic tree was constructed with the protein sequences of conserved cyclin domains. Only p-values for the approximate likelihood ratios (SH-aLRT) of >0.5 (50%) are indicated. Scale bars correspond to 0.5 amino acid substitutions per site. Gene name or ID number is indicated. ANID, *Aspergillus nidulans*; FGSG, *Fusarium graminearum*; MGG, *Magnaporthe oryzae*; NCU, *Neurospora crassa*; Sc, *Saccharomyces cerevisiae*; Sp, *Schizosaccharomyces pombe*; SS1G, *Sclerotinia sclerotiorum*. (B) Expression profile of these four cyclins during sexual reproduction (FG5) and infection of barley spikes (FG1). The expression data of RMA treatment were downloaded from Plant Expression Database (PLEXdb) (www.plexdb.org). Mean and standard deviation were calculated from data of three biological replicates. hpf, hours post-fertilization; hpi, hours post-inoculation.
(PDF)

S8 Fig Phylogeny and alignment of fungal Aurora kinases. (A) A phylogenetic tree of fungal Aurora kinases constructed with the kinase domain sequences. Each branch was marked with the p-values of >0.5 of approximate likelihood ratios (SH-aLRT). Scale bar corresponds to 0.2 amino acid substitutions per site. (B) Multiple sequence alignment shows the amino acid replacement at the position equivalent to G198 of human Aurora-A (red box). Hs, *Homo sapiens*; Fg, *Fusarium graminearum*; Sc, *Saccharomyces cerevisiae*; Sp, *Schizosaccharomyces pombe*.
(PDF)

S1 Table DON production in the *cdc2A* and *cdc2B* mutants and their complemented transformants. (DOCX)

S2 Table Orthologs of mitotic protein kinases in *Fusarium graminearum*. (XLSX)
