# Supplementary figures and images for: Two Cdc2 Kinase Genes with Distinct Functions in Vegetative and Infectious Hyphae in Fusarium graminearum
Source: PLoS Pathog. 2015 Jun 17;11(6):e1004913. doi: 10.1371/journal.ppat.1004913 (PMC4470668; doi:10.1371/journal.ppat.1004913)

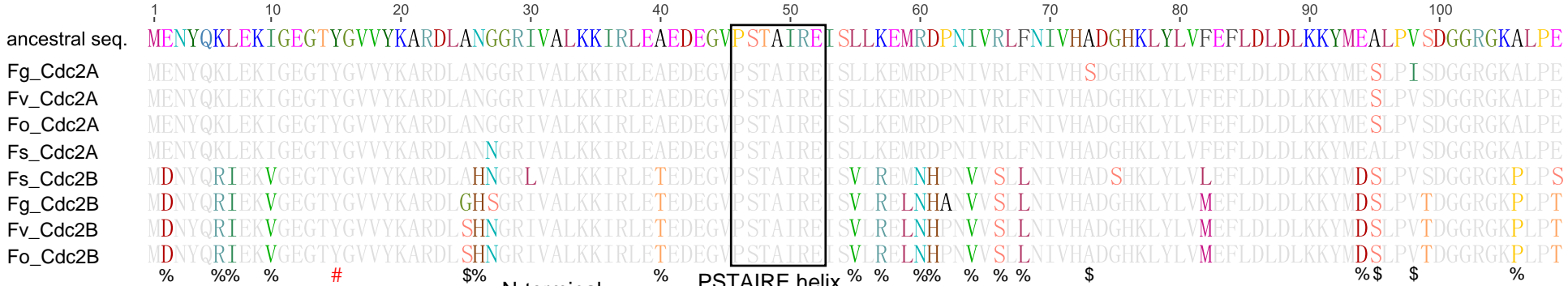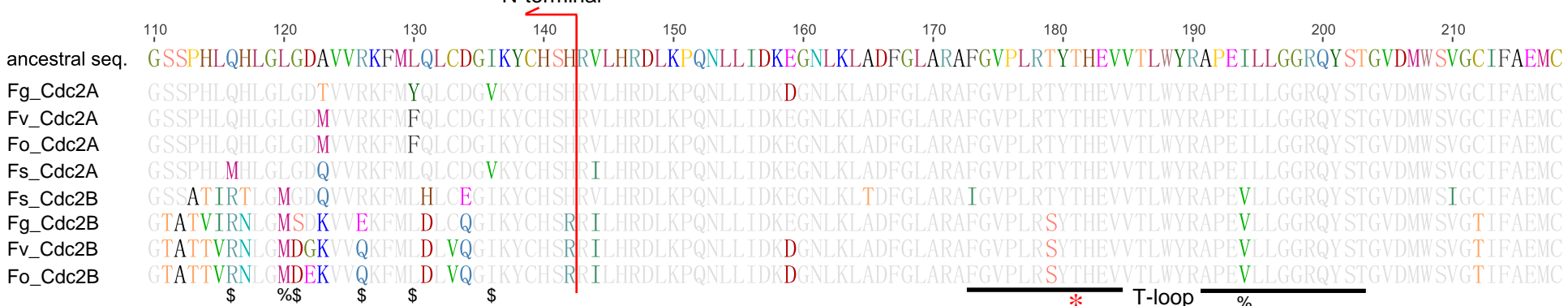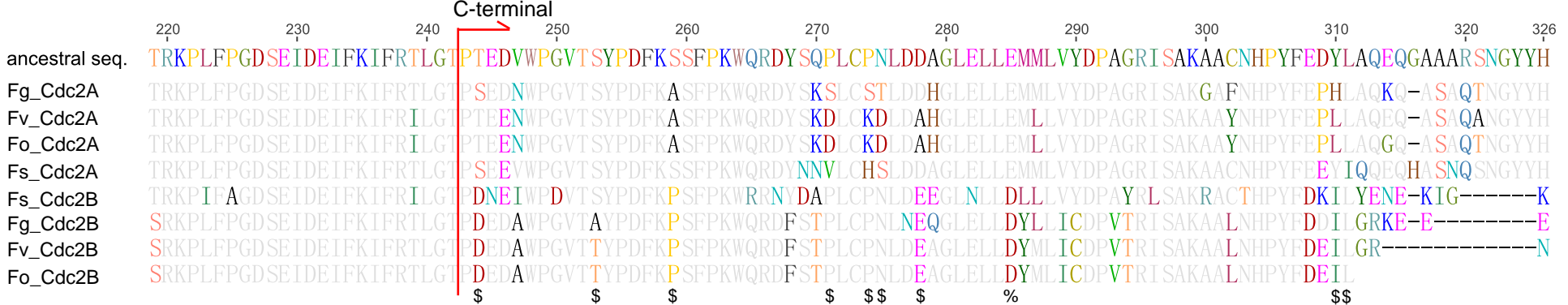

Supplement: S1 Fig — The ancestral sequence reconstructed by the FASTML Server (http://fastml.tau.ac.il/) was shown above as the reference. Residues different from the reference are highlighted in color. The PSTAIRE helix and the T-loop are key landmark regions of Cdc2. ‘#’ and ‘*’ symbols mark the inhibitory (Y15) and activating (T181) phosphorylation sites, respectively. ‘$’ and ‘%’ symbols indicate amino acid sites with type I (highly conserved in one co-ortholog but variable in the other) and type II (highly conserved within both co-orthologs but diverged between them) functional divergence [73] at p > 0.6. Red vertical lines mark the N- and C-terminal regions of Cdc2A and Cdc2B as depicted in Fig 7A. (PDF) [file ppat.1004913.s001.pdf]

● Cdc2A  
● Cdc2B

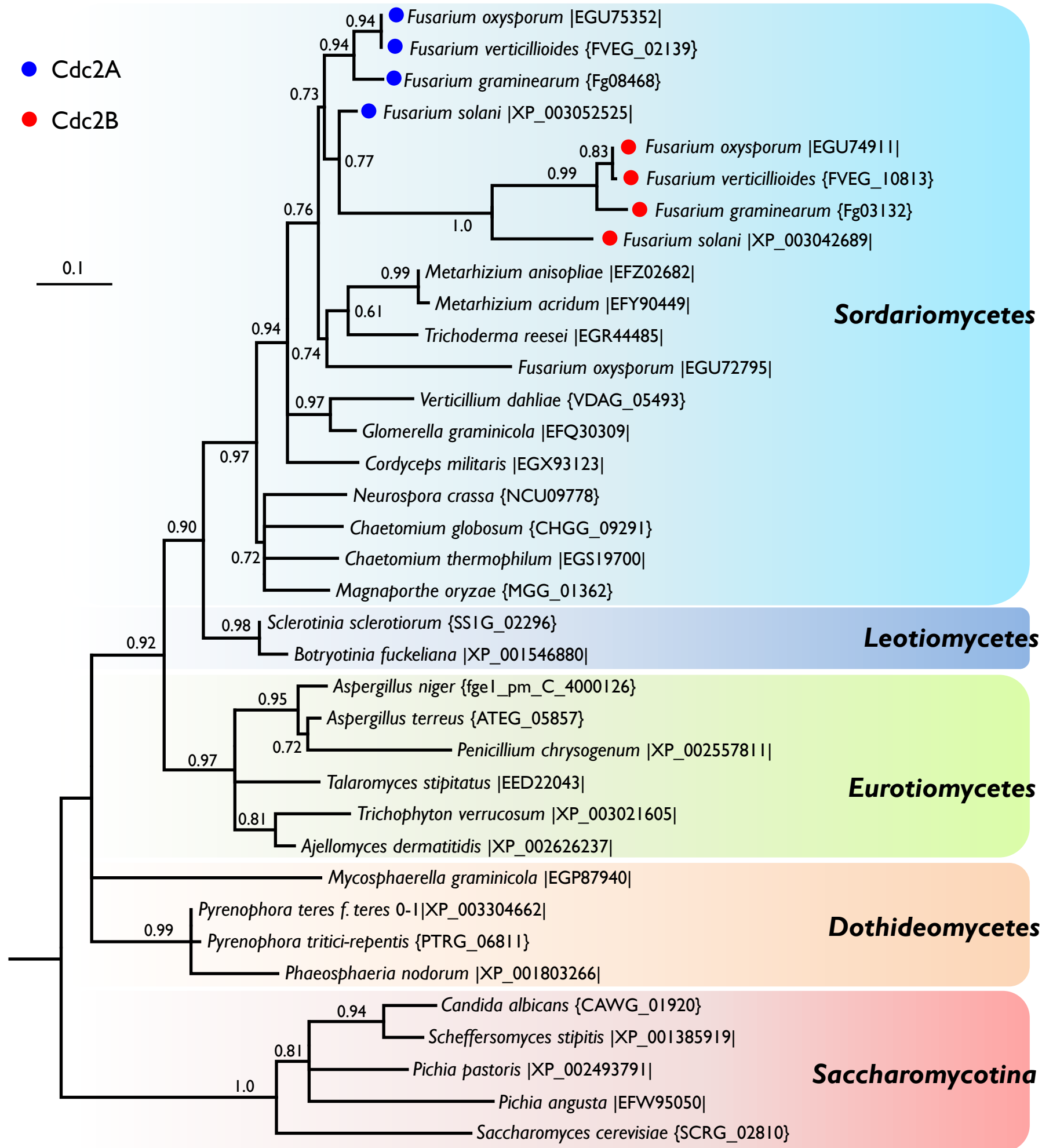

Supplement: S2 Fig — The phylogenetic tree was constructed with the protein sequences of catalytic domains. Each branch was marked with the p-values of approximate likelihood ratios (SH-aLRT). Branches with p-values less than 0.5 have been collapsed. Scale bar corresponds to 0.1 amino acid substitutions per site. F. oxysporum has many lineage-specific genomic regions [33] and is the only fungus with three Cdc2 orthologs. (PDF) [file ppat.1004913.s002.pdf]

**A**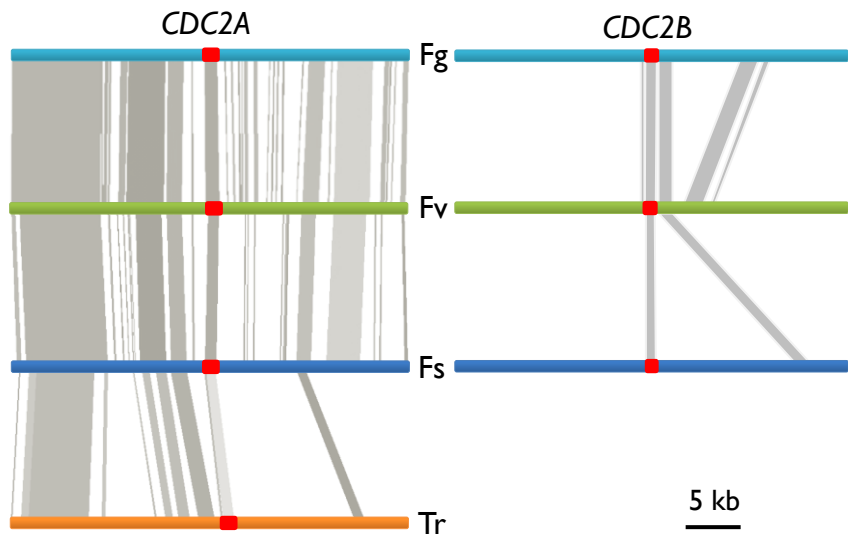**B**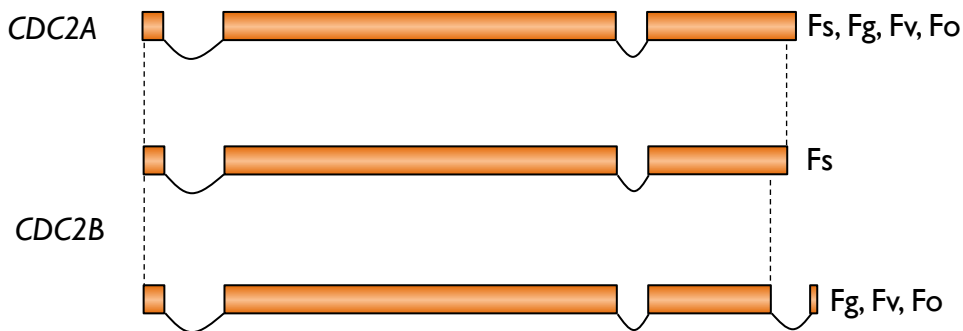

Supplement: S3 Fig — (A) Synteny of the CDC2A and CDC2B loci and their 40-kb flanking sequences. Collinear orthologous relationships are represented by gray bands. Fusarium oxysporum shares a high sequence identity with F. verticillioides in this region. (B) Intron positions in the CDC2A and CDC2B genes. Dashed lines mark the homologous region. Fg, Fusarium graminearum; Fo, F. oxysporum; Fv, F. verticillioides; Fs, F. solani; Tr, Trichoderma reesei. (PDF) [file ppat.1004913.s003.pdf]

**A**

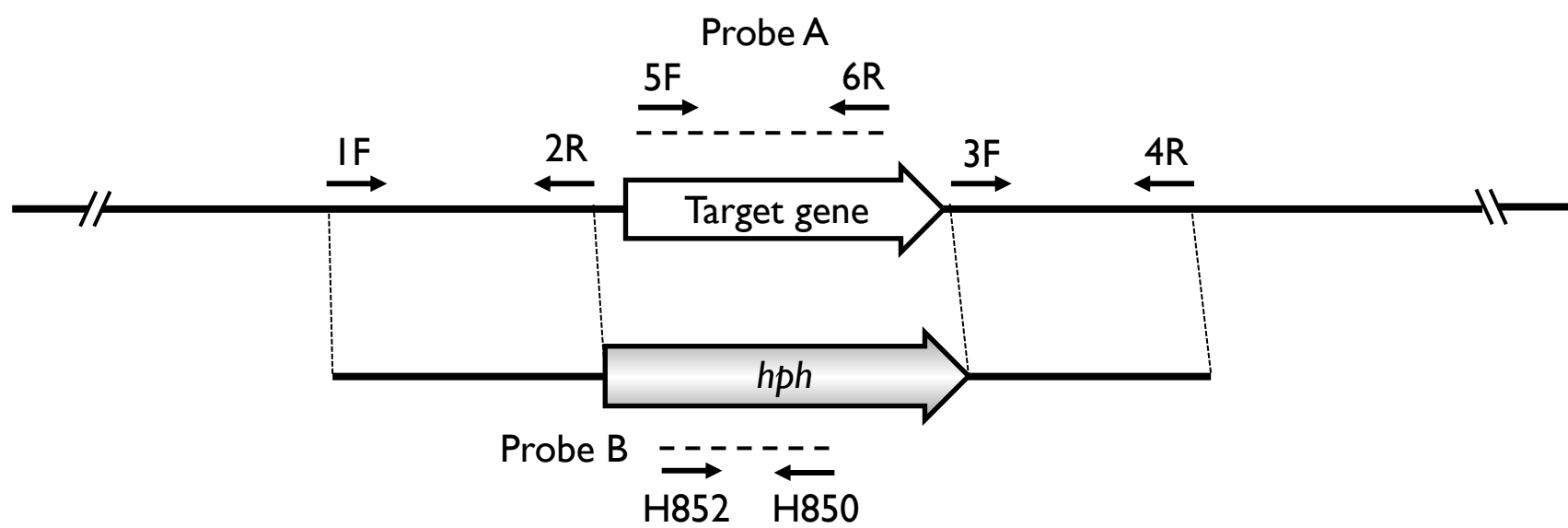

**B**

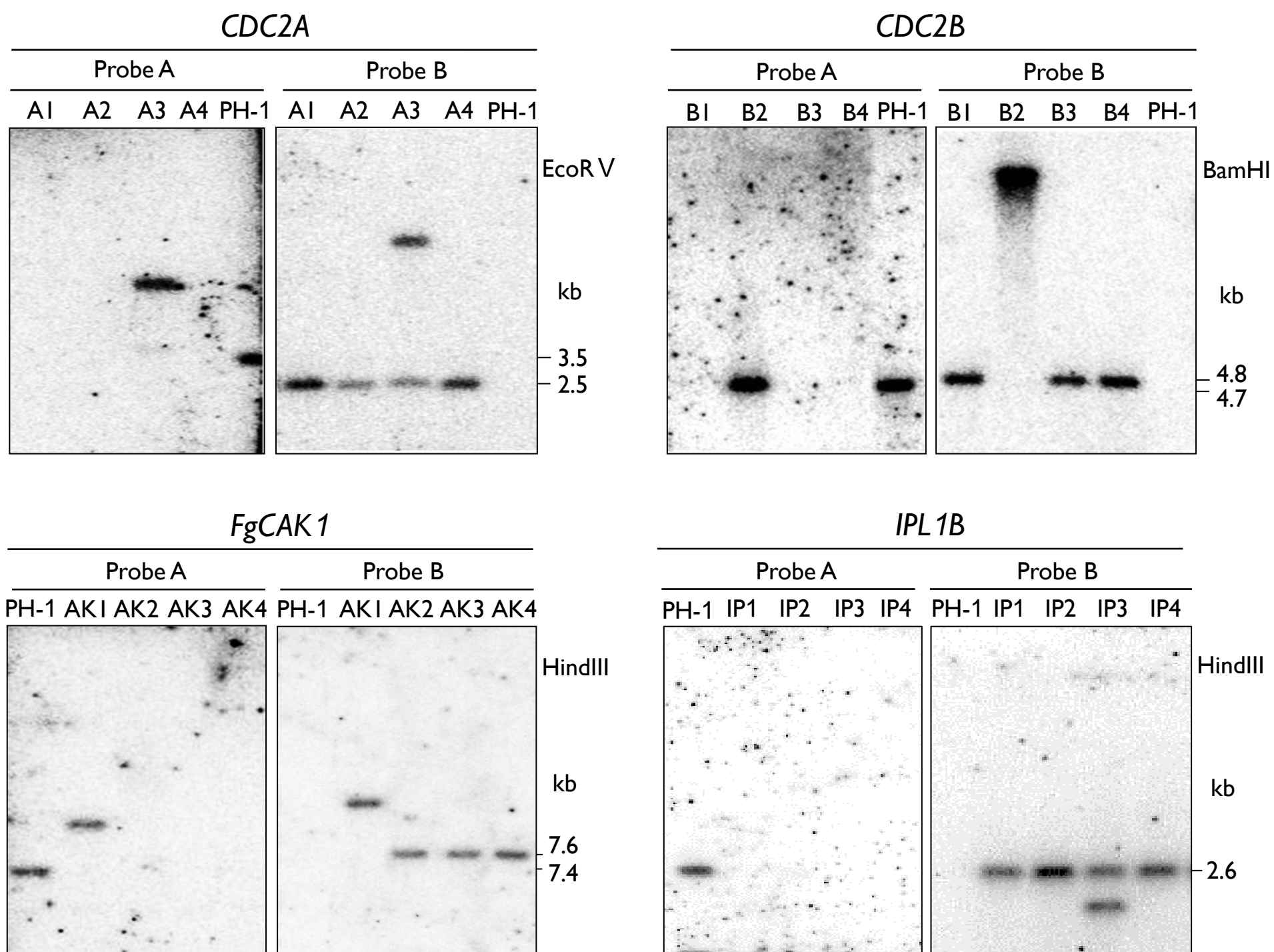

Supplement: S4 Fig — (A) The split-marker approach used to replace the target gene with the hygromycin phosphotransferase (hph) cassette. Probe A and probe B are PCR products amplified with primers 5F/6R and H852/H850, respectively. (B) Southern blots of restriction enzyme-digested genomic DNA of PH-1 (WT) and mutants hybridized with probe A (left) and probe B (right). The restriction enzyme used was marked. (PDF) [file ppat.1004913.s004.pdf]

**A**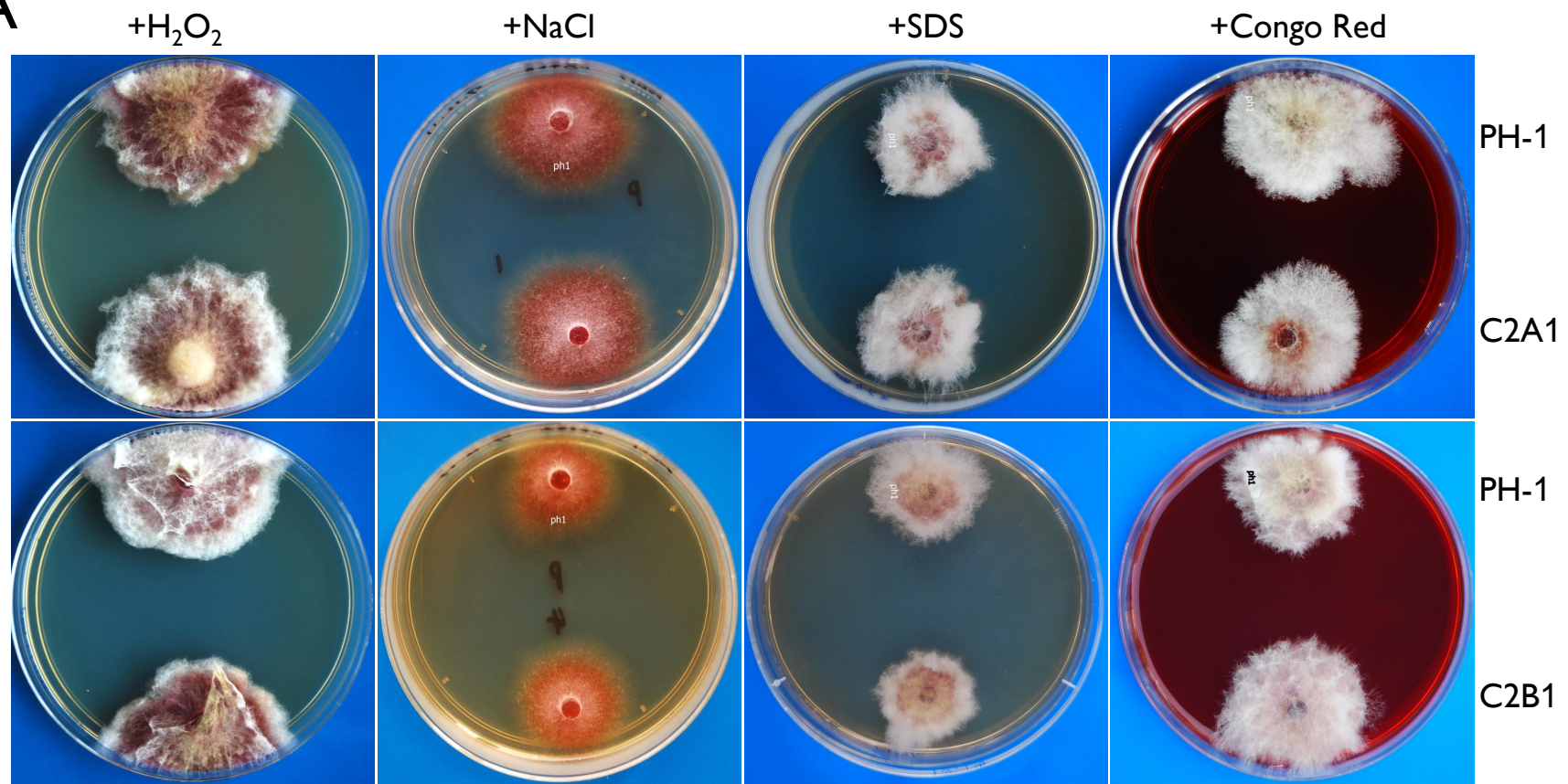**B**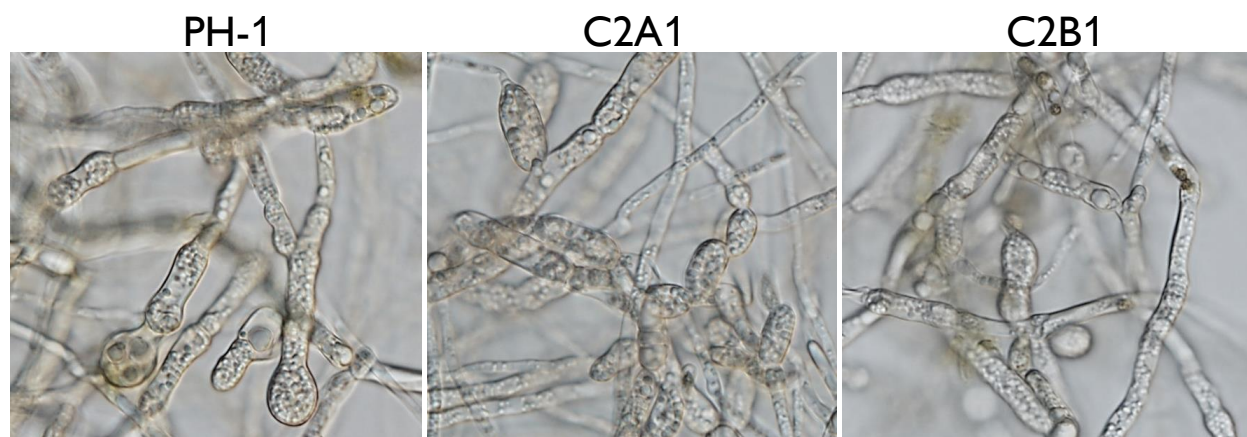

Supplement: S5 Fig — (A) Responses of the cdc2A and cdc2B mutants to oxidative, hyperosmotic, membrane, and cell wall stresses. Four-day old colonies formed by the wild type (PH-1) and cdc2A (C2A1) or cdc2B (C2B1) deletion mutants on PDA with 0.05% H2O2, 0.7 M NaCl, 0.01% SDS, or 200 mg/ml Congo Red. (B) Hyphal morphology of PH-1 and the cdc2A and cdc2B mutants in trichothecene biosynthesis induction (TBI) liquid culture after 72 h of incubation at 25°C. (PDF) [file ppat.1004913.s005.pdf]

C2A-N4

C2B-N2

DIC

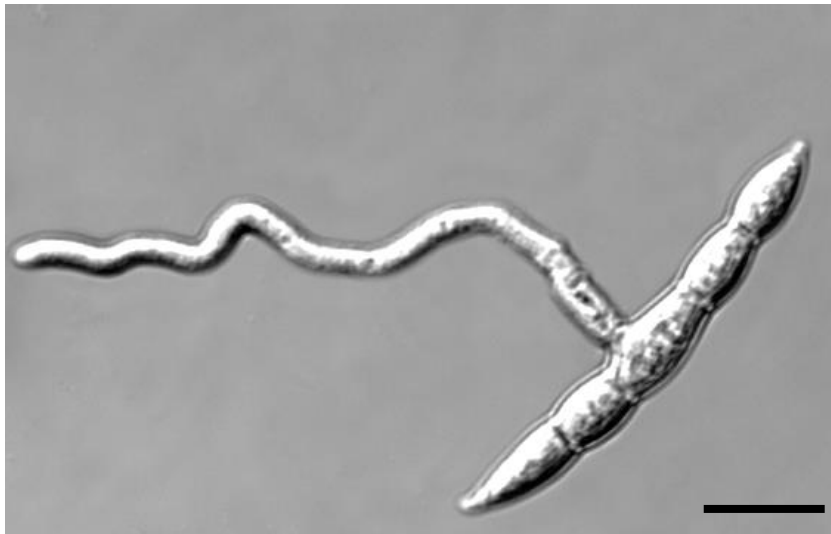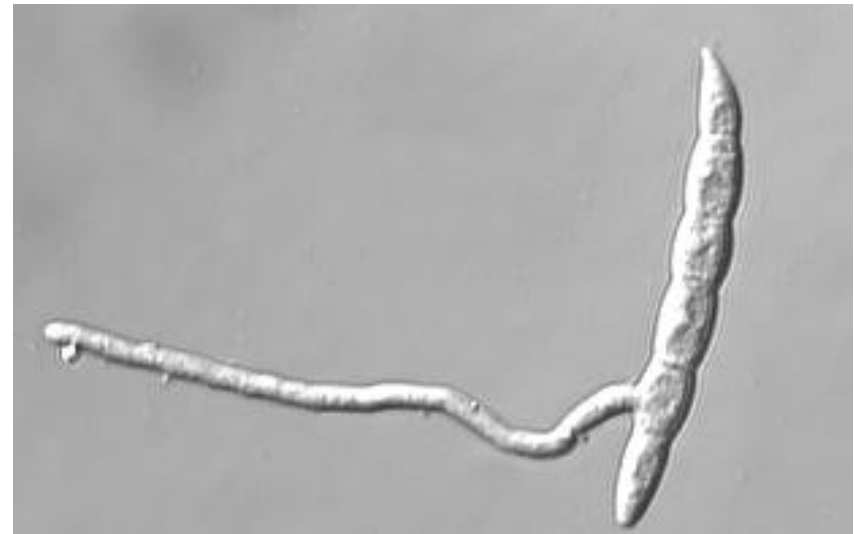

Hoechst

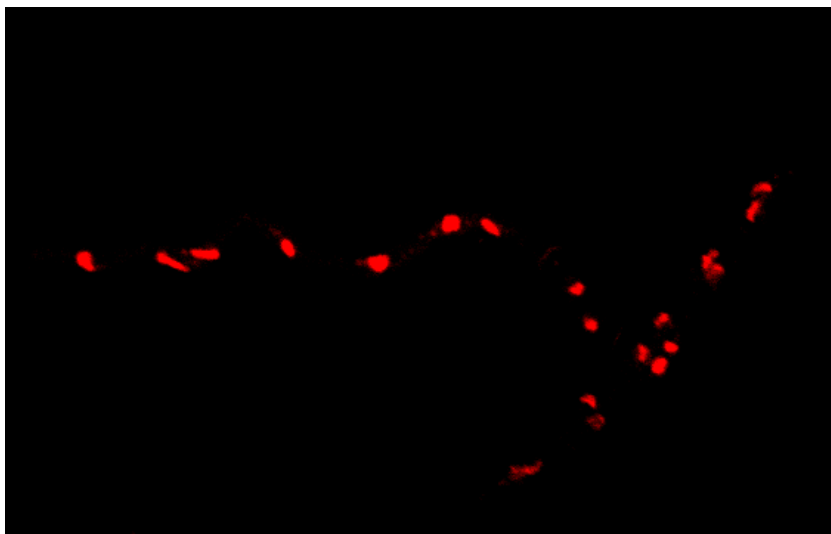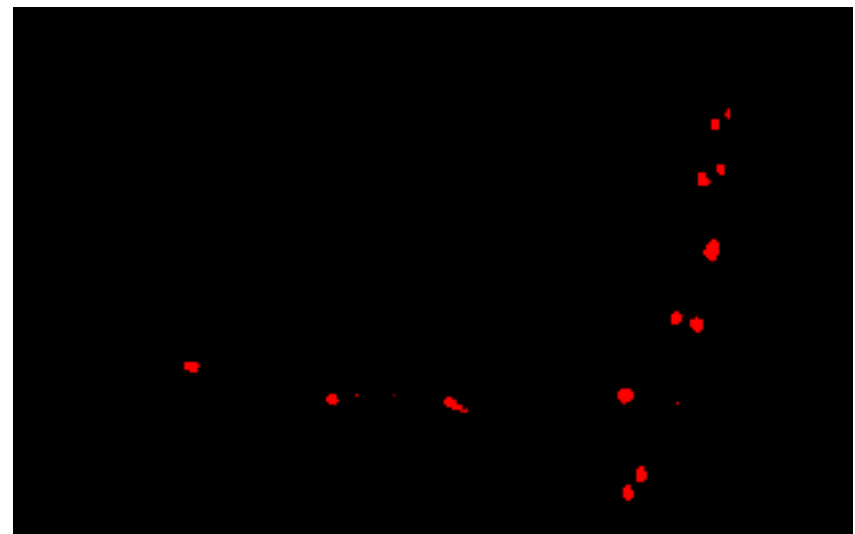

GFP

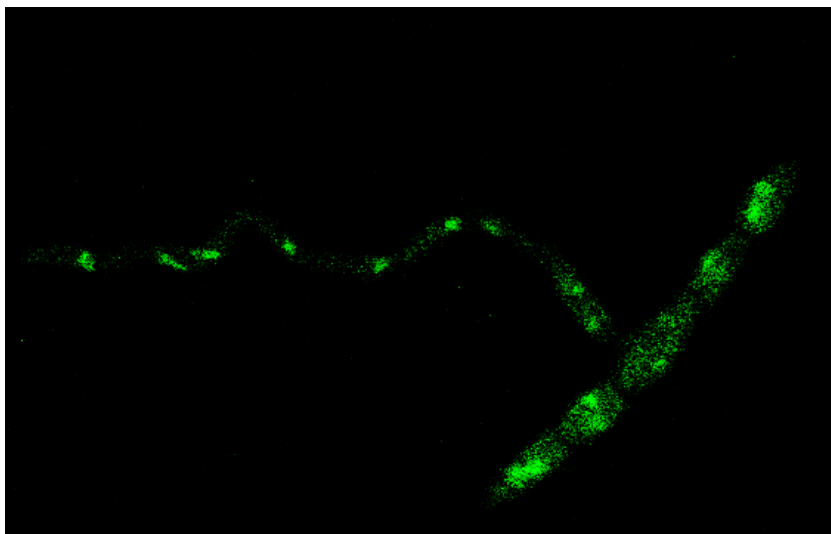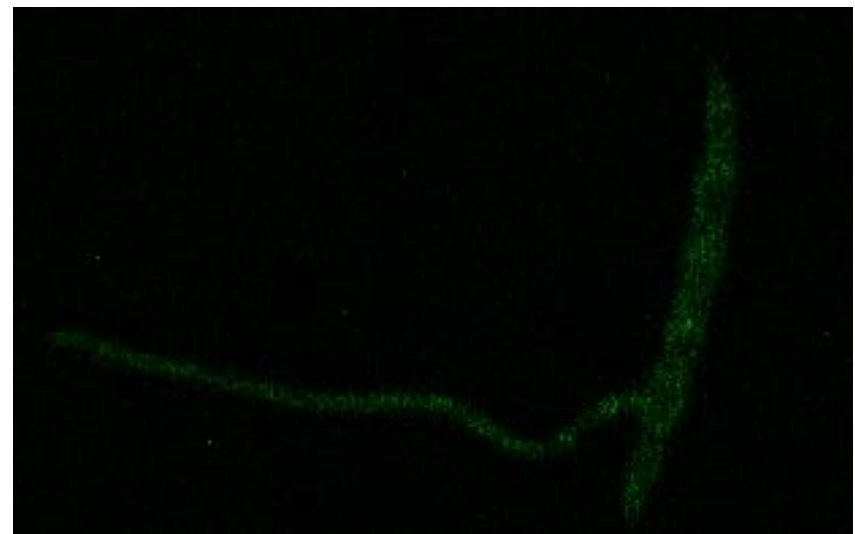

Merged

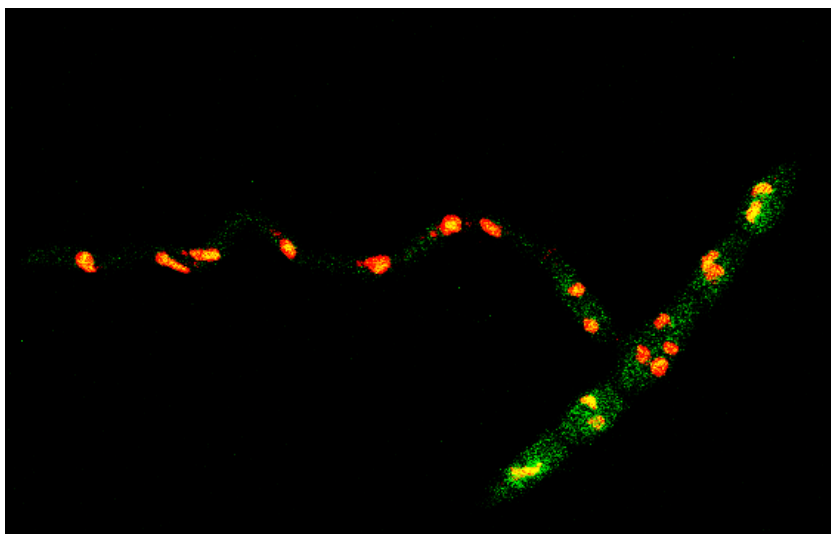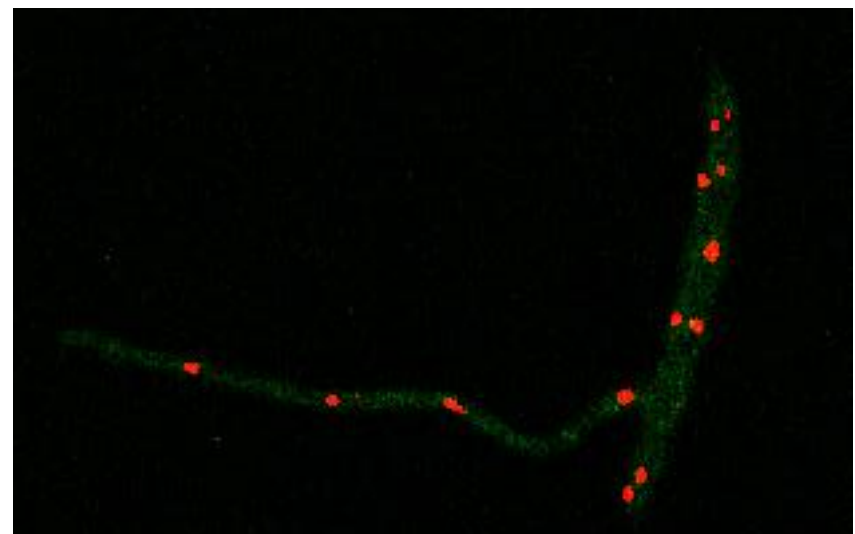

Supplement: S6 Fig — Germlings (8 h) of the CDC2A-GFP (C2A-N4) and CDC2B-GFP (C2B-N2) transformants were examined by differential interference contrast (DIC) and confocal microscopy after staining with Hoechst. Bar = 20 μm. (PDF) [file ppat.1004913.s006.pdf]

**A**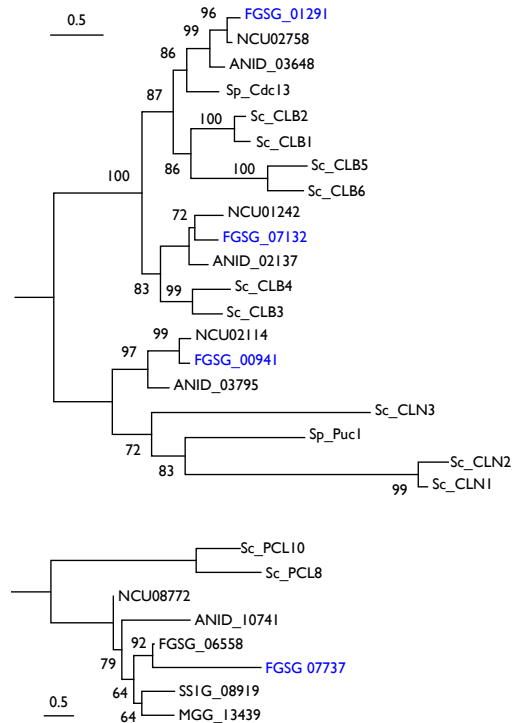**B**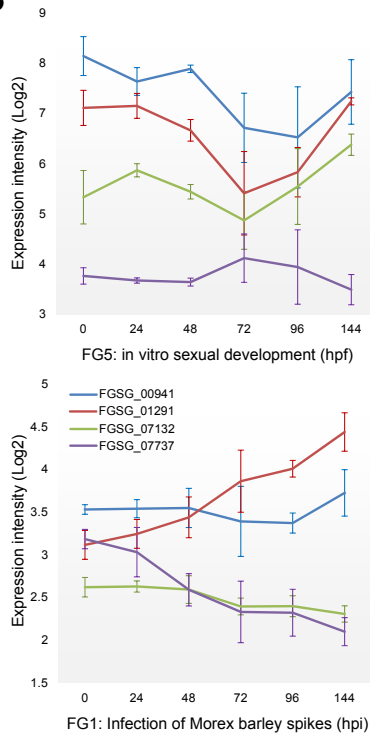

Supplement: S7 Fig — (A) The maximum likelihood tree of orthologs of three putative Cdc2-cyclins FGSG_01291, FGSG_07132, and FGSG_00941 and one extra PCL8/PCL10-like cyclin FGSG_07737. The phylogenetic tree was constructed with the protein sequences of conserved cyclin domains. Only p-values for the approximate likelihood ratios (SH-aLRT) of >0.5 (50%) are indicated. Scale bars correspond to 0.5 amino acid substitutions per site. Gene name or ID number is indicated. ANID, Aspergillus nidulans; FGSG, Fusarium graminearum; MGG, Magnaporthe oryzae; NCU, Neurospora crassa; Sc, Saccharomyces cerevisiae; Sp, Schizosaccharomyces pombe; SS1G, Sclerotinia sclerotiorum. (B) Expression profile of these four cyclins during sexual reproduction (FG5) and infection of barley spikes (FG1). The expression data of RMA treatment were downloaded from Plant Expression Database (PLEXdb) (www.plexdb.org). Mean and standard deviation were calculated from data of three biological replicates. hpf, hours post-fertilization; hpi, hours post-inoculation. (PDF) [file ppat.1004913.s007.pdf]
