# Supplementary material for: Two Cdc2 Kinase Genes with Distinct Functions in Vegetative and Infectious Hyphae in Fusarium graminearum
Source: PLoS Pathog. 2015 Jun 17;11(6):e1004913. doi: 10.1371/journal.ppat.1004913 (PMC4470668; doi:10.1371/journal.ppat.1004913)
Supplement: S8 Fig — (A) A phylogenetic tree of fungal Aurora kinases constructed with the kinase domain sequences. Each branch was marked with the p-values of >0.5 of approximate likelihood ratios (SH-aLRT). Scale bar corresponds to 0.2 amino acid substitutions per site. (B) Multiple sequence alignment shows the amino acid replacement at the position equivalent to G198 of human Aurora-A (red box). Hs, Homo sapiens; Fg, Fusarium graminearum; Sc, Saccharomyces cerevisiae; Sp, Schizosaccharomyces pombe. (PDF) [file ppat.1004913.s008.pdf]

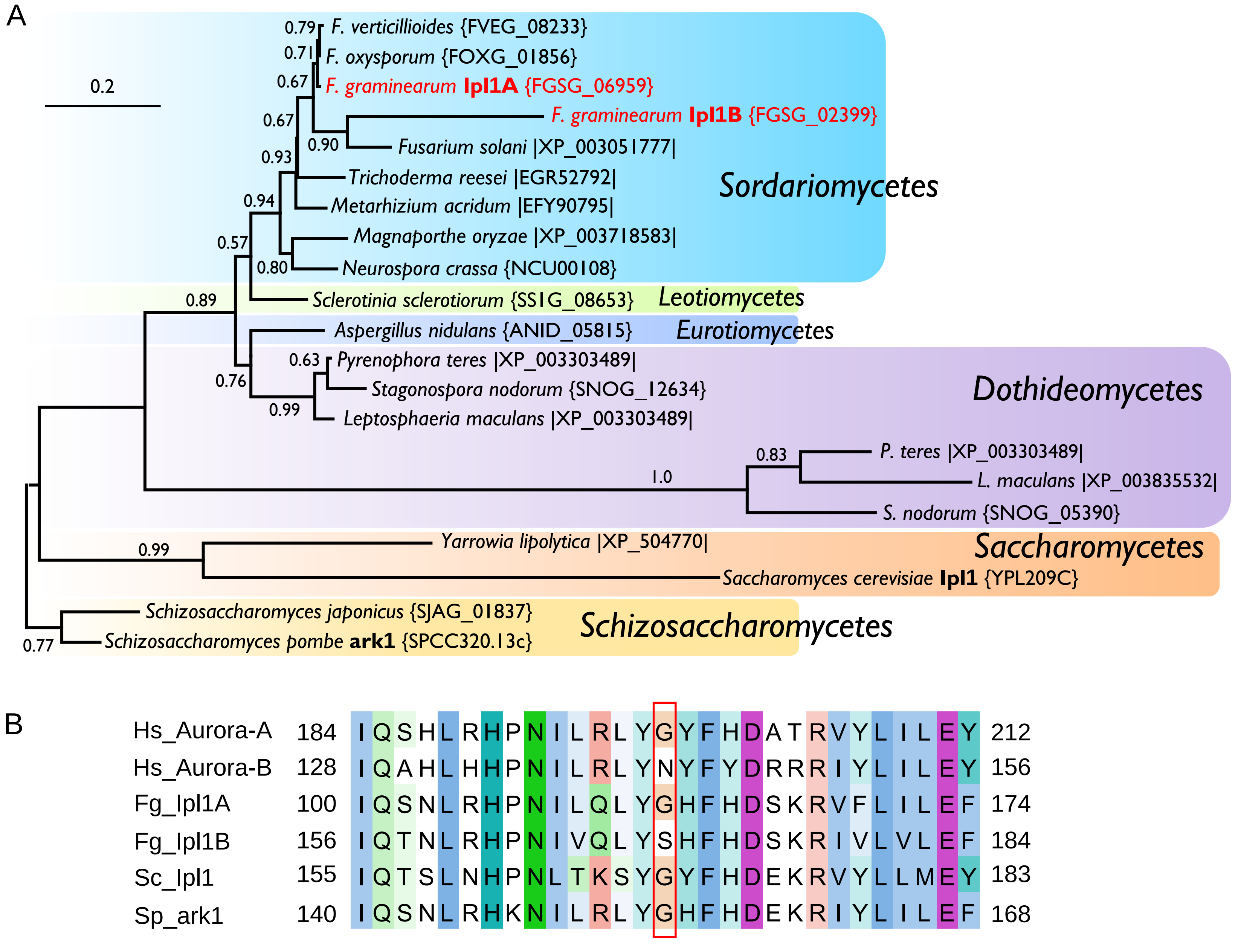
A
0.2
0.79 F. verticillioides {FVEG_08233}
0.71 F. oxysporum {FOXG_01856}
0.67 F. graminearum Ipl1A {FGSG_06959}
0.67
F. graminearum Ipl1B {FGSG_02399}
0.90 Fusarium solani |XP_003051777|
0.93
Trichoderma reesei |EGR52792|
0.94
Metarhizium acridum |EFY90795|
0.57
Magnaporthe oryzae |XP_003718583|
0.80 Neurospora crassa {NCU00108}
Sordariomycetes
0.89
Sclerotinia sclerotiorum {SS1G_08653} Leotiomycetes
Aspergillus nidulans {ANID_05815} Eurotiomycetes
0.63 Pyrenophora teres |XP_003303489|
0.76
Stagonospora nodorum {SNOG_12634}
0.99 Leptosphaeria maculans |XP_003303489|
Dothideomycetes
0.83 P. teres |XP_003303489|
1.0
L. maculans |XP_003835532|
S. nodorum {SNOG_05390}
0.99 Yarrowia lipolytica |XP_504770|
Saccharomycetes
Saccharomyces cerevisiae Ipl1 {YPL209C}
Schizosaccharomyces japonicus {SJAG_01837}
0.77 Schizosaccharomyces pombe ark1 {SPCC320.13c}
Schizosaccharomycetes
B
Hs_Aurora-A 184 I Q S H L R H P N I L R L Y G Y F H D A T R V Y L I L E Y 212
Hs_Aurora-B 128 I Q A H L H H P N I L R L Y N Y F Y D R R R I Y L I L E Y 156
Fg_Ipl1A 100 I Q S N L R H P N I L Q L Y G H F H D S K R V F L I L E F 174
Fg_Ipl1B 156 I Q T N L R H P N I V Q L Y S H F H D S K R I V L V L E F 184
Sc_Ipl1 155 I Q T S L N H P N L T K S Y G Y F H D E K R V Y L L M E Y 183
Sp_ark1 140 I Q S N L R H K N I L R L Y G H F H D E K R I Y L I L E F 168
