# Supplementary material for: Two Cdc2 Kinase Genes with Distinct Functions in Vegetative and Infectious Hyphae in Fusarium graminearum
Source: PLoS Pathog. 2015 Jun 17;11(6):e1004913. doi: 10.1371/journal.ppat.1004913 (PMC4470668; doi:10.1371/journal.ppat.1004913)
Supplement: S1 Table — (DOCX) [file ppat.1004913.s009.docx]

**Table S1. DON production in the *cdc2A* and *cdc2B* mutants and their complemented transformants**

| **Strains** | **DON production (ppm)** | |
| --- | --- | --- |
|  | liquid TBI cultures* | Rice grain cultures |
| PH-1 (WT) | 668.5±195^A^ | 1782.1±519.2^A^ |
| C2A1 (*Δcdc2A*) | 917.9±273.5^A^ | 1803.6±294.7^A^ |
| C2A-N4 (*cdc2A* comp.) | 675.3±66.7^A^ | 1771.5±501.5^A^ |
| C2B1 (*Δcdc2B*) | 691.3±54.7^A^ | 1816.9±468.9^A^ |
| C2B-N1 (*cdc2B* comp.) | 890.6±150.9^A^ | 1913.3±441.9^A^ |

* DON production was assayed with liquid trichothecene biosynthesis induction (TBI) or rice grain cultures. Mean and standard deviations were calculated with results from three biological replicates. Data were analyzed with the one-way analysis of variance (ANOVA). The same letter indicated no significant difference in DON production (P = 0.05).
